# Digital and In-Person Mindfulness-Based Interventions for University Students’ Mental Health: A Systematic Review of Randomized Controlled Trials

**DOI:** 10.3390/healthcare14131875

**Published:** 2026-06-26

**Authors:** Sharmistha Roy, Amar Kanekar, Ashis Kumar Biswas, Manoj Sharma

**Affiliations:** 1 Department of Social and Behavioral Health, School of Public Health, University of Nevada, Las Vegas, NV 89119, USA; 2 School of Counseling, Human Performance and Rehabilitation, University of Arkansas at Little Rock, Little Rock, AR 72204, USA; 3 Department of Management Information Systems, Lee Business School, University of Nevada, Las Vegas, NV 89154, USA; 4 Department of Internal Medicine, Kirk Kerkorian School of Medicine at UNLV, University of Nevada, Las Vegas, NV 89102, USA

**Keywords:** mindfulness-based interventions, meditation, digital interventions, in-person interventions, stress, anxiety, depression, university students, randomized controlled trials

## Abstract

**Highlights:**

**What are the main findings?**

- Mindfulness-based interventions significantly reduce depression and anxiety among university students, while effects on stress are mixed and context-dependent.
- Digital and technology-enhanced interventions are widely used and scalable, but show variability in adherence and consistency of outcomes.

**What are the implications of the main findings?**

- Mindfulness interventions offer accessible, non-pharmacological strategies for improving student mental health and can be integrated into university health systems.
- Future research should focus on optimizing intervention design, improving engagement in digital formats, and assessing long-term effectiveness across diverse populations.

**Abstract:**

**Background/Objectives:** University students commonly experience psychological distress driven by academic demands, social transitions, and financial pressures. Mindfulness-based interventions have emerged as scalable approaches to improve mental health. However, evidence comparing their effectiveness across delivery formats remains limited. This systematic review aimed to evaluate the effectiveness of mindfulness-based interventions in reducing stress, anxiety, and depression and to compare outcomes across in-person, digital, and hybrid modalities. **Methods**: This review followed PRISMA 2020 guidelines and included randomized controlled trials (RCTs) published between January 2020 and December 2025 on mindfulness-based interventions among university students aged 18 years and older. Intervention duration ranged from 3 days to 12 weeks, with most lasting 4 to 8 weeks, and outcomes included validated measures of stress, anxiety, or depression. Literature research was conducted in PubMed, PsycINFO, CINAHL, Scopus, and Web of Science, and two reviewers independently screened studies, extracted data, and assessed methodological quality using the Joanna Briggs Institute checklist. **Results:** A total of 24 RCTs were included, with the highest representation from the United States and China (*n* = 4 each), followed by the United Kingdom and Canada. Mindfulness-based interventions demonstrated consistent reductions in depression and generally positive effects on anxiety, while effects on stress were more variable. Digital interventions demonstrated effectiveness comparable to in-person programs, though outcomes varied by intervention structure and level of guidance. **Conclusions:** Mindfulness-based interventions are effective in improving mental health among university students, particularly for depression and anxiety. Multi-week programs and guided digital delivery appear to enhance effectiveness and scalability.

## 1. Introduction

Mental health problems among university and college students have become a critical global public health concern [1]. Young adults enrolled in higher education face a wide range of developmental, academic, financial, and social pressures during a period marked by rapid life transitions [2]. These pressures include demanding coursework, competitive expectations, economic instability, shifting social networks, and ongoing uncertainty about future careers [3]. At the same time, many students are navigating identity formation and increasing autonomy. Together, these challenges create conditions that heighten vulnerability to psychological distress. International evidence suggests that approximately one-third of university students experience clinically significant anxiety or depressive symptoms, and substantial proportions report elevated stress during their academic programs [2,4]. These outcomes are associated with impaired academic performance, absenteeism, reduced retention, sleep disturbances, substance misuse, self-harm, suicidal ideation, and increased likelihood of long-term mental health consequences extending into adulthood [2,3].

Recent epidemiological evidence indicates that the prevalence and severity of mental health problems among university students continue to rise globally. Reports from the Healthy Minds Study show that the proportion of students reporting positive mental health declined from 51% in 2013–2014 to 38% in 2023–2024 [2,5,6]. In the same body of evidence, approximately 38% of students reported depressive symptoms and 34% reported anxiety symptoms, while serious suicidal thoughts reached 13%, and suicide planning rose to 6% [6,7]. Large surveys further indicate that 76% of students experience moderate to high levels of stress, 49% report loneliness, and fewer than half receive counseling services despite nearly half screening positive for anxiety or depression [7,8].

Additional evidence suggests that nearly 60% of students experience overwhelming anxiety, 35.1% report that anxiety interferes with academic performance, and 34.6% report having been diagnosed with an anxiety disorder [7,9]. Globally, anxiety prevalence among university students has been estimated at about 31%, with some European estimates rising as high as 55% [10,11]. Together, these findings underscore the urgency of developing effective, scalable, and accessible interventions for student mental health.

The burden of psychological distress among students intensified during and after the COVID-19 pandemic [12]. The abrupt transition to online learning, disruptions to campus life, reduced peer interaction, financial strain, and uncertainty regarding educational and future career pathways created heightened emotional stress for many students [2,13]. At the same time, universities struggled to meet increased demand for psychological services. Traditional counseling systems often face structural limitations, including long wait times, insufficient staffing, and inadequate reach. Help-seeking is also impeded by stigma, scheduling barriers, limited mental health literacy, and concerns about confidentiality [14]. This treatment gap remains substantial, with nearly two-thirds of students experiencing mental health problems not seeking or receiving adequate support [14]. These barriers have increased interest in flexible and scalable mental health strategies that complement campus counseling. Internet- and mobile-based interventions (IMIs) have emerged as promising options because they can be accessed anonymously, independent of time and place, and at lower cost than many traditional services [14].

Such formats may be particularly suitable for university students, who often face competing academic demands and may prefer self-guided or low-stigma modes of support. Evidence further suggests that IMIs can reach students who might otherwise avoid formal treatment and, when guided, may perform comparably to face-to-face approaches in some contexts [3,14]. Within this context, mindfulness-based interventions have increasingly been adapted into digital and hybrid delivery formats, enhancing their potential for scalable implementation in university settings. Mindfulness is commonly defined as an intentional, present-moment, and nonjudgmental awareness of internal and external experiences [4]. It is cultivated through practices such as breathing meditation, body scans, mindful movement, and open monitoring of thoughts and emotions.

Within this growing digital mental health landscape, mindfulness- and meditation-based interventions have gained increasing attention. Several structured interventions are based on this approach, especially Mindfulness-Based Stress Reduction (MBSR) and Mindfulness-Based Cognitive Therapy (MBCT). MBSR emphasizes awareness and acceptance of present experiences to improve self-regulation under stress, whereas MBCT integrates mindfulness with cognitive therapy principles to help individuals recognize and interrupt maladaptive thought patterns linked to anxiety and depression [13,15]. Acceptance and Commitment Therapy (ACT), which is also used in some digital mindfulness programs, extends this approach by emphasizing psychological flexibility and committed action in line with personal values [15,16].

The theoretical rationale underlying these interventions is strong. Mindfulness-based approaches are thought to reduce psychological distress by improving attentional control, decreasing rumination, enhancing emotional regulation, and promoting a more adaptive relationship to negative thoughts and emotions [4,17]. Recent studies suggest that changes in negative automatic thoughts may be one of the mechanisms through which MBCT reduces anxiety and stress among college students [13]. In one trial, changes in negative automatic thoughts accounted for 28% of the intervention effect on trait anxiety and 45% of the effect on chronic stress, indicating that mindfulness-based programs may work not only through relaxation but also through cognitive change processes [13]. Other work has shown that baseline mindfulness may influence intervention response, suggesting that individual differences in pre-intervention psychological functioning are important when evaluating program effectiveness [4].

A growing body of randomized controlled trials has examined mindfulness-based interventions among university students. Brief online mindfulness programs have shown beneficial effects on depression, rumination, and trait anxiety [4]. Internet-based and mobile-supported mindfulness interventions have also shown improvements in mindfulness, well-being, anxiety, depression, and stress, although effect sizes and adherence vary across programs and populations [3,14,15]. Additional randomized trials suggest benefits for stress, depression, insomnia, and anxiety-related outcomes, although not all trials find consistent effects across all mental health domains [18,19,20]. However, variability in intervention structure, duration, and delivery modality has contributed to inconsistencies in observed effects, particularly for stress-related outcomes.

Several systematic reviews have previously evaluated mindfulness-based interventions among university students. Wong et al. (2018) highlighted the growing evidence supporting mindfulness-based approaches and emphasized intervention implementation, effectiveness, and safety considerations in higher education settings [21]. Dawson et al. (2020) synthesized randomized controlled trials and reported beneficial effects on psychological distress, depression, anxiety, mindfulness, self-compassion, and well-being among university students [22]. More recently, Chen et al. (2023) conducted a systematic review and meta-analysis of mobile mindfulness meditation interventions among university students and found significant improvements in stress, anxiety, well-being, and mindfulness, although no significant effects were observed for depression or resilience [23]. Similarly, Zuo et al. (2023) reported that mindfulness-based interventions were associated with improvements in depression, anxiety, stress, and sleep quality among university students [24]. Collectively, these reviews established an important evidence base supporting the use of mindfulness-based interventions in higher education settings.

Despite the growing evidence base, several important knowledge gaps remain. First, most existing systematic reviews, including Wong et al. (2018), Dawson et al. (2020), Chen et al. (2023), and Zuo et al. (2023), synthesized evidence generated largely before or during the early stages of the rapid expansion of digital and hybrid mindfulness interventions following the COVID-19 pandemic [21,22,23,24]. Consequently, the effectiveness, feasibility, acceptability, and implementation characteristics of contemporary delivery modalities remain incompletely understood. Second, relatively few evidence syntheses have directly compared in-person, digital, and hybrid mindfulness interventions, despite the possibility that delivery format may influence engagement, adherence, accessibility, and intervention effectiveness. Third, substantial heterogeneity persists regarding intervention duration, intensity, technological components, and levels of facilitator involvement, making it difficult to identify characteristics associated with optimal outcomes.

Fourth, emerging mechanisms of action, including mindfulness, psychological flexibility, emotional regulation, self-compassion, and changes in negative automatic thoughts, have received limited attention in previous reviews despite their importance for intervention refinement and theory development [13,14]. Finally, the post-pandemic period has witnessed unprecedented growth in scalable digital mental health approaches for university students, creating a need for an updated synthesis of recent randomized evidence. The year 2020 was selected as the starting point for this review because it coincided with the onset of the COVID-19 pandemic and the accelerated adoption of digital mental health services, online learning environments, and technology-assisted mindfulness interventions within higher education settings [13,15].

The review question was structured according to the PICOS framework. The population comprised university and college students aged 18 years or older. The interventions included mindfulness and meditation-based programs delivered through in-person, digital, or hybrid modalities. The comparator conditions included waitlist controls, usual care, psychoeducation, attention controls, or other non-mindfulness interventions. The primary outcomes were stress, anxiety, and depression, assessed using validated psychometric instruments. Only randomized controlled trials were included.

Therefore, this systematic review aimed to synthesize randomized controlled trials published between 2020 and 2025 examining digital, in-person, and hybrid mindfulness-based interventions among university students. Specifically, this review sought to evaluate intervention characteristics, delivery modalities, mental health outcomes, and potential mechanisms of action relevant to contemporary university settings. By focusing on recent evidence generated during a period of rapid technological and service-delivery transformation, this review provides an updated understanding of how mindfulness-based interventions can support student mental health in modern higher education environments.

## 2. Materials and Methods

### 2.1. Protocol and Reporting Standards

This systematic review was conducted in accordance with the Preferred Reporting Items for Systematic Reviews and Meta-Analyses (PRISMA 2020) statement to ensure transparent and reproducible reporting of the review process [25]. The protocol was prospectively registered on the Open Science Framework (OSF) on 8 December 2025 (https://doi.org/10.17605/OSF.IO/JP9QX; OSF registration number: JP9QX; accessed 8 December 2025). All methodological procedures, including the eligibility criteria, information sources, search strategy, study selection process, data extraction plan, critical appraisal approach, and synthesis strategy, established a priori in the registered protocol before the review was conducted.

### 2.2. Eligibility Criteria (PICOS Framework)

Eligibility criteria were defined using the Population, Intervention, Comparator, Outcomes, and Study Design (PICOS) framework. The population of interest included university or college students aged 18 years or older who were enrolled in undergraduate or graduate programs. Studies involving health, medical, or other professional students (e.g., nursing, pharmacy, or allied health trainees) were excluded. Professional students were excluded because their academic experiences, clinical placement requirements, licensing examinations, and profession-specific stressors may differ substantially from those of the general university student population. Restricting eligibility to non-professional student populations was intended to improve population comparability and reduce potential confounding related to specialized educational environments. Studies were eligible if they evaluated mindfulness- or meditation-based interventions, including but not limited to Mindfulness-Based Stress Reduction (MBSR), Mindfulness-Based Cognitive Therapy (MBCT), breathing meditation, mindful awareness practices, yoga-based meditation, compassion-focused meditation, digital mindfulness applications, web-based mindfulness interventions, mindfulness-CBT hybrid programs, or peer-supported meditation interventions (Table 1).

Eligible comparator conditions included waitlist controls, no-treatment controls, usual care, attention controls, psychoeducation, or alternative non-mindfulness interventions. Included studies were required to report at least one validated psychological outcome related to stress, anxiety, and/or depression, measured with standardized instruments such as the Perceived Stress Scale (PSS), Depression Anxiety Stress Scales (DASS-21 or DASS-42), Generalized Anxiety Disorder Scale (GAD-7), Beck Anxiety Inventory (BAI), State–Trait Anxiety Inventory (STAI), Patient Health Questionnaire (PHQ-8 or PHQ-9), or Hospital Anxiety and Depression Scale (HADS).

The primary outcomes of interest were stress, anxiety, and depression. Studies reporting related psychological constructs, such as psychological distress, emotional regulation, or sleep-related outcomes, were considered when relevant to the interpretation of these primary outcomes. Only randomized controlled trials (RCTs) were considered eligible. Additional eligibility criteria required studies to be published in peer-reviewed journals, written in English, and published between 1 January 2020, and 31 December 2025. Studies were excluded if they were observational, qualitative, cross-sectional, case-based, pilot, or feasibility studies without an appropriate control group, non-mindfulness interventions such as exercise-only programs, or studies that did not report psychological outcomes related to stress, anxiety, or depression (Table 2).

### 2.3. Information Sources and Search Strategy

A comprehensive literature search was conducted in five electronic databases: PubMed/MEDLINE, PsycINFO, CINAHL, Scopus, and Web of Science. The search period covered 1 January 2020, through 31 December 2025, to capture recent evidence reflecting both the post-COVID transformation in university mental health and the rapid expansion of digital and hybrid mindfulness interventions during this period. The search strategy combined controlled vocabulary terms and free-text keywords using Boolean operators. In PubMed, Medical Subject Headings (MeSH) were used where appropriate; in PsycINFO and CINAHL, thesaurus and subject-heading terms were applied and in Scopus and Web of Science, title, abstract, and topic-field searches were used. Search concepts focused on four core domains: mindfulness/meditation, student population, psychological outcomes, and trial design. Representative search terms included mindfulness, meditation, mindfulness-based, MBSR, MBCT, breathing meditation, mindful breathing, body scan, university students, college students, undergraduate, graduate, stress, anxiety, depression, mental health, randomized, and randomized controlled trial. Database-specific filters were applied to limit results to English-language articles published from 2020 to 2025.

### 2.4. Study Selection

All retrieved records were exported to a reference management system for organization and to remove duplicates. A total of 604 records were identified through database searching. After removing 45 duplicate records, 559 unique records remained for screening. Two reviewers (SR & AB) independently screened titles and abstracts against the predefined eligibility criteria. During this stage, 429 records were excluded for not meeting the inclusion criteria, including studies that were irrelevant to the research question, did not involve university or college student populations, did not evaluate mindfulness- or meditation-based interventions, did not report relevant mental health outcomes, or did not use an eligible study design. Following title and abstract screening, 130 full-text articles were assessed for eligibility. All reports were successfully retrieved (*n* = 0 not retrieved). Full-text articles were independently reviewed by two reviewers using the predefined inclusion and exclusion criteria.

**Figure 1:**
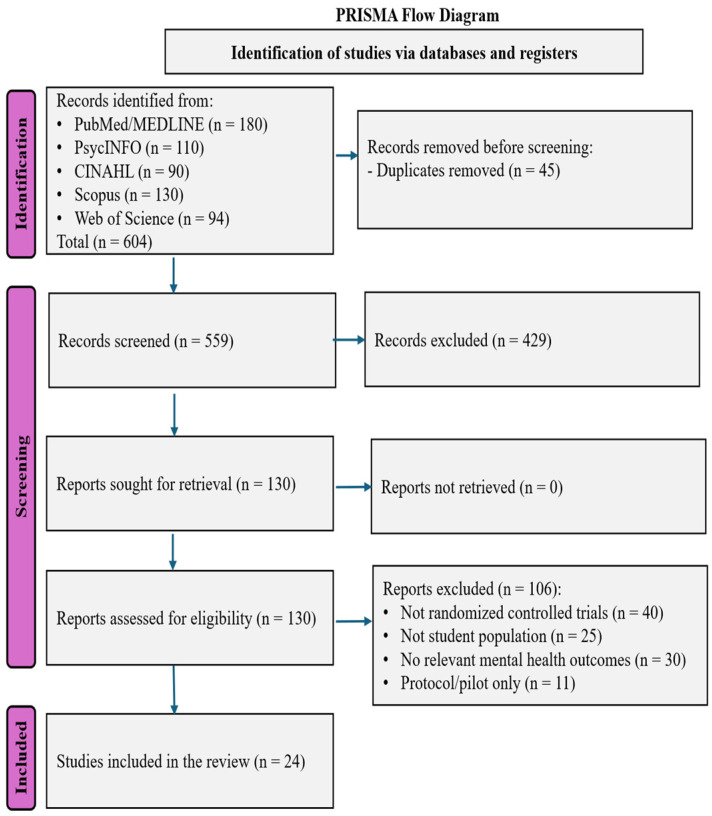
PRISMA Flow diagram.

At the full-text stage, 106 studies were excluded for the following reasons: non-randomized or uncontrolled study design (*n* = 40), insufficient methodological detail or lack of clear randomization procedures (*n* = 40), inclusion of non-student populations (*n* = 25), absence of relevant mental health outcomes (*n* = 30), and protocol or pilot-only publications without post-intervention results (*n* = 11). Any disagreements at both the screening and full-text stages were resolved through discussion and consensus. A total of 24 studies met all eligibility criteria and were included in the qualitative synthesis. The study selection process is presented in the PRISMA 2020 flow diagram (Figure 1).

### 2.5. Data Extraction

A structured and standardized data extraction form was developed in accordance with PRISMA 2020 recommendations for transparent review reporting [25]. The form was pilot-tested on three included studies to ensure clarity, completeness, and consistency before being applied to the full sample of included trials. Two reviewers independently extracted data from all eligible studies, and discrepancies were resolved through discussion and consensus, with third-party consultation available if needed. The extracted data included study characteristics, such as author, publication year, country, institutional setting, sample size, demographic characteristics, and study design, intervention characteristics, such as the type of mindfulness or meditation intervention, duration in weeks, session frequency, delivery modality, facilitator credentials, and core intervention components; comparator characteristics, such as waitlist, usual care, psychoeducation, or active control conditions; and outcome information, including the validated psychometric measures used to assess stress, anxiety, and depression. In addition, the extraction included effectiveness findings, such as statistical significance, reported effect sizes, and between-group or within-group comparisons, as well as quality appraisal data derived from the critical appraisal tool.

### 2.6. Risk of Bias Assessment

The methodological quality of the included studies was assessed using the Joanna Briggs Institute (JBI) critical appraisal checklist for randomized controlled trials [26]. This checklist evaluates key domains of internal validity, including the adequacy of randomization, allocation concealment, baseline group comparability, blinding of participants, blinding of intervention providers, blinding of outcome assessors, completeness of follow-up, consistency of treatment delivery, analysis by assigned groups (e.g., intention-to-treat analysis), reliability and consistency of outcome measurement, and appropriateness of statistical analyses.

Each domain was rated as Yes, No, Unclear, or Not Applicable. Given the behavioral nature of mindfulness- and meditation-based interventions, blinding of participants and intervention providers was often not feasible; therefore, these domains were frequently rated as No or Unclear. Risk-of-bias assessments were conducted independently by two reviewers (SR & AB), and any discrepancies were resolved through discussion and consensus. In interpreting the findings of this review, studies with greater methodological limitations, particularly those related to allocation concealment, blinding, and incomplete follow-up, were interpreted with caution to avoid overestimating intervention effects.

To provide an overall assessment of methodological quality, studies meeting ≥ 80% of applicable JBI criteria were considered high quality, studies meeting 60–79% of criteria were considered moderate quality, and studies meeting < 60% of criteria were considered low quality. These overall quality assessments were used to aid interpretation of findings but were not used as exclusion criteria.

### 2.7. Synthesis of Results

Given the anticipated heterogeneity in intervention content, delivery modality, duration, session intensity, participant characteristics, outcome measures, and reporting formats, the findings were synthesized using a narrative synthesis approach rather than a statistical meta-analysis. This approach was considered appropriate due to substantial variability across studies, which limited the feasibility of quantitative pooling.

Studies were first organized according to the primary mental health outcomes they reported, specifically stress, anxiety, and depression, to enable structured comparison across key domains of psychological distress among university students. In cases where studies did not report these outcomes as distinct constructs, closely related measures such as but not limited to overall psychological distress, emotional regulation, or sleep-related outcomes were considered and interpreted within the corresponding domains to ensure a comprehensive and inclusive synthesis of the evidence. Within each outcome domain, the synthesis evaluated the direction of effects, statistical significance, and magnitude of change using reported effect size indicators, including Cohen’s d, Hedges’ g, beta coefficients, mean differences, and confidence intervals where available. When standardized effect sizes were not reported, findings were interpreted based on statistical significance and the direction of change. Particular attention was paid to determining whether interventions demonstrated not only statistically significant improvements but also clinically meaningful changes.

To explore potential sources of variation in intervention effectiveness, studies were further compared based on delivery modality, including in-person, digital, and hybrid/blended formats, as well as therapeutic frameworks, such as Mindfulness-Based Stress Reduction (MBSR), Mindfulness-Based Cognitive Therapy (MBCT), breath-focused meditation, yoga-based mindfulness practices, app-based interventions, and web-based cognitive-behavioral mindfulness programs. This comparative approach facilitated a deeper understanding of how different intervention characteristics may influence mental health outcomes.

In addition, the synthesis considered the cultural and geographic contexts of the included studies by examining trials conducted across diverse countries and higher education settings. This perspective supported the interpretation of the extent to which mindfulness- and meditation-based interventions may be generalizable across student populations with varying cultural, academic, and healthcare environments. A formal meta-analysis was not conducted due to substantial heterogeneity in intervention structures, outcome measures, study designs, and reporting approaches, which precluded meaningful statistical aggregation. Consistent with PRISMA 2020 guidance, narrative synthesis was therefore deemed the most appropriate and methodologically robust strategy for integrating the available evidence.

## 3. Results

### 3.1. Study Characteristics

Overall, the included studies demonstrated substantial variation in geographic location, sample size, intervention modality, and intervention duration, reflecting the diversity of mindfulness-based interventions implemented among university students. The characteristics of the included studies are summarized in Table 3. The 24 randomized controlled trials were conducted across a wide range of geographical regions, including North America, Europe, Asia, South America, Oceania, and Turkey, indicating strong global representation. Sample sizes varied considerably, ranging from 40 to 616 participants, with most studies focusing on undergraduate or general university student populations. Across studies, participants were primarily college or university students, although a small number included both undergraduate and graduate students.

**Figure 2:**
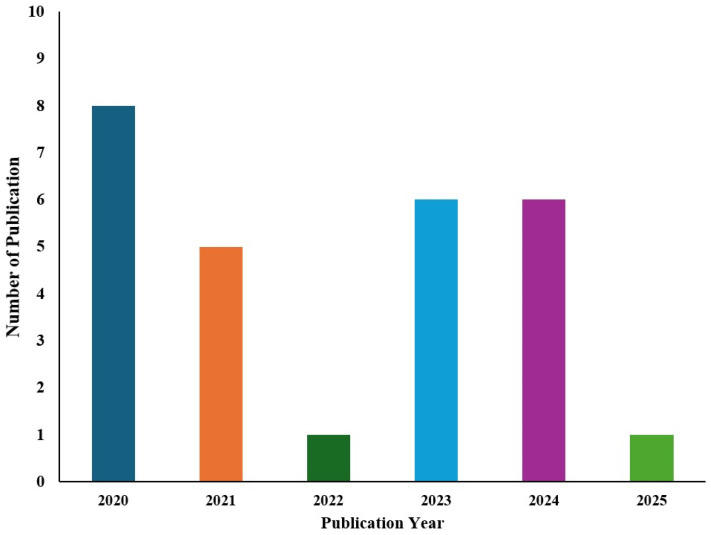
Distribution of included studies by year of publication (2020–2025).

**Figure 3:**
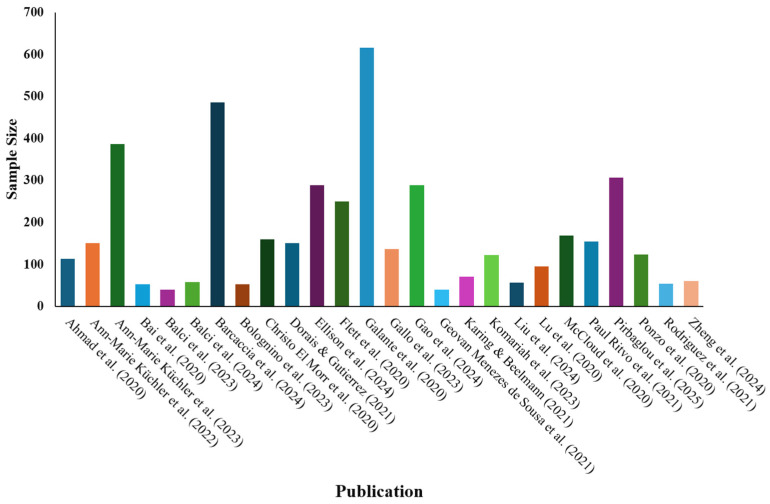
Sample size distribution across included studies [1,4,14,15,16,17,18,19,20,25,26,27,28,29,30,31,32,33,34,35,36,37,38,39,40,41].

**Figure 4:**
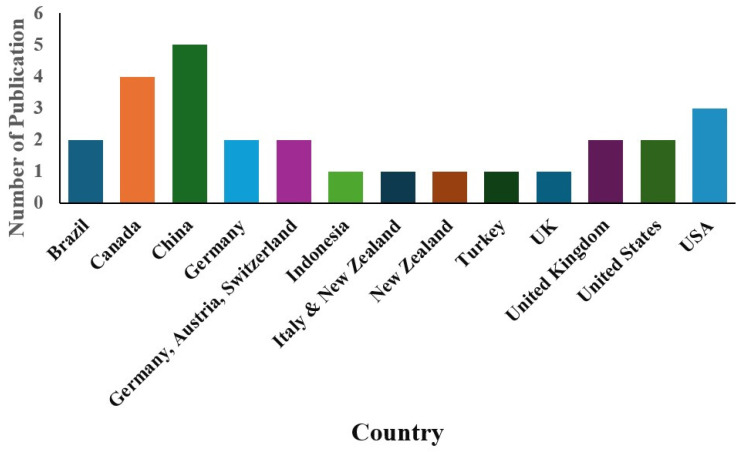
Geographic distribution of included studies by country.

The distribution of included studies by year of publication is presented in Figure 2, showing a higher concentration of studies in 2020 and a resurgence in 2023 to 2024, with fewer studies in 2022 and 2025. Variation in sample sizes across studies is illustrated in Figure 3, highlighting heterogeneity in study scale. The geographic distribution of included studies is shown in Figure 4, demonstrating broad international representation, with the highest number of studies conducted in China, followed by Canada, the United States, and several European countries.

This figure illustrates the number of included randomized controlled trials published each year, showing a higher concentration of studies in 2020 and a resurgence in 2023–2024, with fewer studies in 2022 and 2025.

This figure shows variation in sample sizes across the included studies, highlighting substantial heterogeneity, ranging from small pilot-scale trials to large multi-participant studies.

This figure shows the number of studies conducted in each country, indicating broad international representation, with the highest number in China, followed by Canada, the United States, and European countries.

### 3.2. Intervention Characteristics

As shown in Table 3, the interventions were predominantly grounded in mindfulness principles and frequently combined with cognitive-behavioral therapy (CBT) components. Many studies implemented mindfulness-based cognitive-behavioral therapy approaches, while others used internet- and mobile-based mindfulness programs, app-based interventions, or traditional in-person mindfulness training. Some studies also employed innovative designs, including virtual reality-based interventions, biofeedback-integrated systems, or comparisons with other approaches such as CBT or relaxation techniques. The diversity of intervention types across the included studies is further illustrated in Figure 3.

### 3.3. Delivery Modes

For descriptive purposes, intervention delivery modalities were categorized as digital, in-person, or hybrid approaches. The delivery modes varied substantially across studies (Table 3). Digital interventions constituted the largest category and were delivered through online or web-based platforms, mobile applications, structured modules, discussion forums, videoconferencing platforms, and self-guided digital programs. Mobile and smartphone applications were also commonly used.

In-person interventions primarily involved face-to-face group-based mindfulness training sessions facilitated by instructors. Hybrid interventions combined digital and face-to-face components, such as online learning modules supplemented with in-class activities or instructor-guided sessions. Additionally, some interventions were self-guided or audio-guided, and a few incorporated advanced technologies such as virtual reality or wearable devices.

### 3.4. Duration of Interventions

The duration of interventions ranged from very brief programs lasting 3 days to longer interventions extending up to 12 weeks (Table 3). However, most studies implemented interventions within a 4 to 8-week timeframe. Short-term interventions included brief intensive formats, while longer programs provided more sustained engagement. Overall, the included mindfulness-based interventions demonstrated considerable diversity in delivery modality, intervention structure, technological integration, and duration, highlighting the heterogeneity of contemporary mindfulness programs for university students.

### 3.5. Intervention Objectives and Models

The objectives and intervention models of the included studies are summarized in Table 4. The primary outcomes targeted across studies were stress, anxiety, and depression, while mindfulness, emotional regulation, self-compassion, resilience, and psychological well-being were examined as related or exploratory outcomes when reported. Across the included studies, interventions were designed to reduce psychological distress and improve mental health and well-being among university students. Many interventions combined mindfulness-based approaches with cognitive-behavioral therapy (CBT) components, such as mindfulness-based cognitive-behavioral therapy [31,42] and Acceptance and Commitment Therapy (ACT) integrated with Mindfulness-Based Stress Reduction (MBSR) [20,32]. In addition, several studies implemented standalone mindfulness interventions, including mindfulness meditation [33], centering meditation [28], and mindfulness breathing practices [37]. Other studies incorporated hybrid or comparative models, such as biofeedback combined with CBT and mindfulness [38], Qigong-based mind–body exercise compared with CBT [34], and virtual reality-based mindfulness interventions [41]. Overall, the findings demonstrate considerable diversity in the intervention models and therapeutic approaches used across the included studies.

### 3.6. Core Components of Interventions

As shown in Table 4, the core components of interventions commonly included guided meditation, breathing exercises, body awareness practices, and psychoeducation. Many programs also incorporated cognitive restructuring, emotional regulation strategies, and reflective exercises, particularly in CBT-integrated interventions [1,29]. Several interventions included interactive and social components, such as peer discussion boards and group videoconferencing [31,42], while others emphasized self-guided practices, including audio-guided meditation and mobile app-based activities [30,35]. Additionally, some studies incorporated innovative elements, such as ecological momentary assessment and wearable biofeedback [38] or immersive virtual reality environments [41].

### 3.7. Session Format and Frequency

For descriptive purposes, interventions were categorized as digital, in-person, or hybrid according to their primary delivery modality. The session formats varied considerably across studies (Table 4), ranging from self-guided modules and mobile applications to structured group sessions and guided interventions. Many interventions used online modules or app-based formats, allowing flexible participation [20,29], whereas others employed in-person group sessions, often led by trained instructors [18,23]. The frequency of sessions also varied, with most studies implementing weekly sessions or modules, while others required daily practice or multiple sessions per week [28,37]. Short-duration interventions included intensive formats such as daily sessions over a few days [33] or brief daily practices over two weeks [30].

### 3.8. Instructor Support and Delivery Platforms

Instructor support ranged from fully guided interventions with trained professionals to self-guided programs with minimal or no support (Table 4). Several studies provided support from certified mindfulness instructors or mental health professionals [1,14], while others used peer counselors or e-coaches [32,40]. In contrast, some interventions were entirely self-guided, particularly those delivered through mobile applications or audio recordings [30,35]. The delivery platforms included web-based platforms, smartphone applications, online modules, traditional in-person formats, and hybrid approaches. A small number of studies also incorporated emerging technologies, such as virtual reality [41].

### 3.9. Study Characteristics and Measurement Approaches

Measurement approaches and outcome instruments used across studies are summarized in Table 5. Across the included randomized controlled trials, a range of validated instruments were used to assess mental health outcomes among university students. Stress was most commonly measured using the Perceived Stress Scale (PSS) or related instruments such as the Perceived Stress Questionnaire (PSQ). Anxiety outcomes were frequently assessed using the Beck Anxiety Inventory (BAI), Generalized Anxiety Disorder-7 (GAD-7), or State–Trait Anxiety Inventory (STAI). Depression outcomes were primarily evaluated using the Patient Health Questionnaire-9 (PHQ-9) or Beck Depression Inventory (BDI). Some studies assessed broader constructs such as psychological distress, rumination, sleep quality, or emotional regulation. The primary outcomes synthesized in this review were stress, anxiety, and depression, whereas related constructs were examined separately as exploratory outcomes when reported.

#### 3.9.1. Effects on Stress Outcomes

Findings related to stress outcomes were heterogeneous across studies (Table 5). Several trials demonstrated statistically significant reductions in perceived stress following mindfulness-based interventions. For instance, Ritvo et al. (2021) [35] reported a significant reduction in perceived stress (B = −2.31, *p* = 0.03), while Küchler et al. (2022) [25] observed a large reduction in stress (d = −0.92). Similarly, Küchler et al. (2023) [16] reported small to moderate improvements in stress (d about 0.25–0.69), and Galante et al. (2020) [14] found a sustained reduction in psychological distress at one-year follow-up (β = −0.22, *p* < 0.001). Additional studies such as Ponzo et al. (2020) [33], Ahmad et al. (2020) [1], and Komariah et al. (2023) [38] also reported significant reductions in stress.

In contrast, some studies did not observe statistically significant changes in stress outcomes. El Morr et al. (2020) [28] reported no significant improvement in perceived stress (B = 0.64, *p* = 0.48), while Balci et al. (2023, 2024) [4,17] also found no meaningful changes in stress levels. De Sousa et al. (2021) [34] reported that stress reduction occurred only within the mindfulness intervention group and was mediated by increases in state mindfulness. Some studies reported outcomes related to stress rather than direct measures of perceived stress. For example, Barcaccia et al. (2024) [39] demonstrated a significant reduction in rumination (F(1483) = 12.05, *p* < 0.001), Bai et al. (2020) [27] showed stabilization of stress-related emotional responses rather than reductions in stress itself, and Liu et al. (2024) [26] reported significant improvements in sleep quality. These findings are presented as related exploratory outcomes and should be interpreted separately from direct stress outcomes.

#### 3.9.2. Effects on Anxiety Outcomes

Most studies reported reductions in anxiety, although the magnitude and consistency of effects varied (Table 5). Strong and statistically significant reductions in anxiety were observed in several trials. El Morr et al. (2020) [28] reported a substantial decrease in anxiety (B = −4.82, *p* = 0.006), while Küchler et al. (2022) [25] reported moderate reductions (d = −0.50). Similarly, Küchler et al. (2023) [16], Ponzo et al. (2020) [33], Komariah et al. (2023) [38], and Liu et al. (2024) [26] demonstrated statistically significant improvements in anxiety symptoms. Barcaccia et al. (2024) [39] also found a significant reduction in trait anxiety (F(1483) = 12.43, *p* < 0.001), and Ellison et al. (2024) [15] reported moderate improvements in anxiety outcomes. However, not all studies demonstrated consistent effects. Ritvo et al. (2021) [35] reported an anxiety reduction that was not statistically significant (B = −2.06, *p* = 0.31). McCloud et al. (2020) [32] found that anxiety was significantly reduced at week 4 (MD = −1.94, *p* = 0.001) but not sustained at week 6 (*p* = 0.09). De Sousa et al. (2021) [34] reported reductions only among individuals with high trait mindfulness. Additionally, some studies, such as Gallo et al. (2023) [19] and Balci et al. (2024) [17], reported limited or non-significant anxiety effects, and in some cases, anxiety was included within broader psychological distress constructs rather than assessed independently.

#### 3.9.3. Effects on Depression Outcomes

Reductions in depressive symptoms were consistently observed across several studies, with many reporting statistically significant improvements (Table 5). El Morr et al. (2020) [28] found a significant reduction in depression (B = −2.21, *p* = 0.01), while Küchler et al. (2022) [25] reported a large effect (d = −0.87). Küchler et al. (2023) [16], Ponzo et al. (2020) [33], Ahmad et al. (2020) [1], Liu et al. (2024) [26], and Komariah et al. (2023) [38] also demonstrated statistically significant reductions in depressive symptoms. McCloud et al. (2020) [32] reported a significant decrease in depressive symptoms at week 6 (MD = −1.56, *p* = 0.006), and Barcaccia et al. (2024) [39] reported a strong reduction in depression (F(1483) = 17.55, *p* < 0.001). In contrast, some studies found no meaningful effects. Ritvo et al. (2021) [35] reported no improvement in depression (B = 0.44, *p* = 0.64), and Balci et al. (2023, 2024) [4,17] also reported non-significant changes. Additionally, some studies did not directly measure depression outcomes, such as de Sousa et al. (2021) [34] while others, including Galante et al. (2020) [14], assessed depression indirectly as part of broader psychological distress measures.

### 3.10. Theoretical Frameworks and Intervention Models

**Figure 5:**
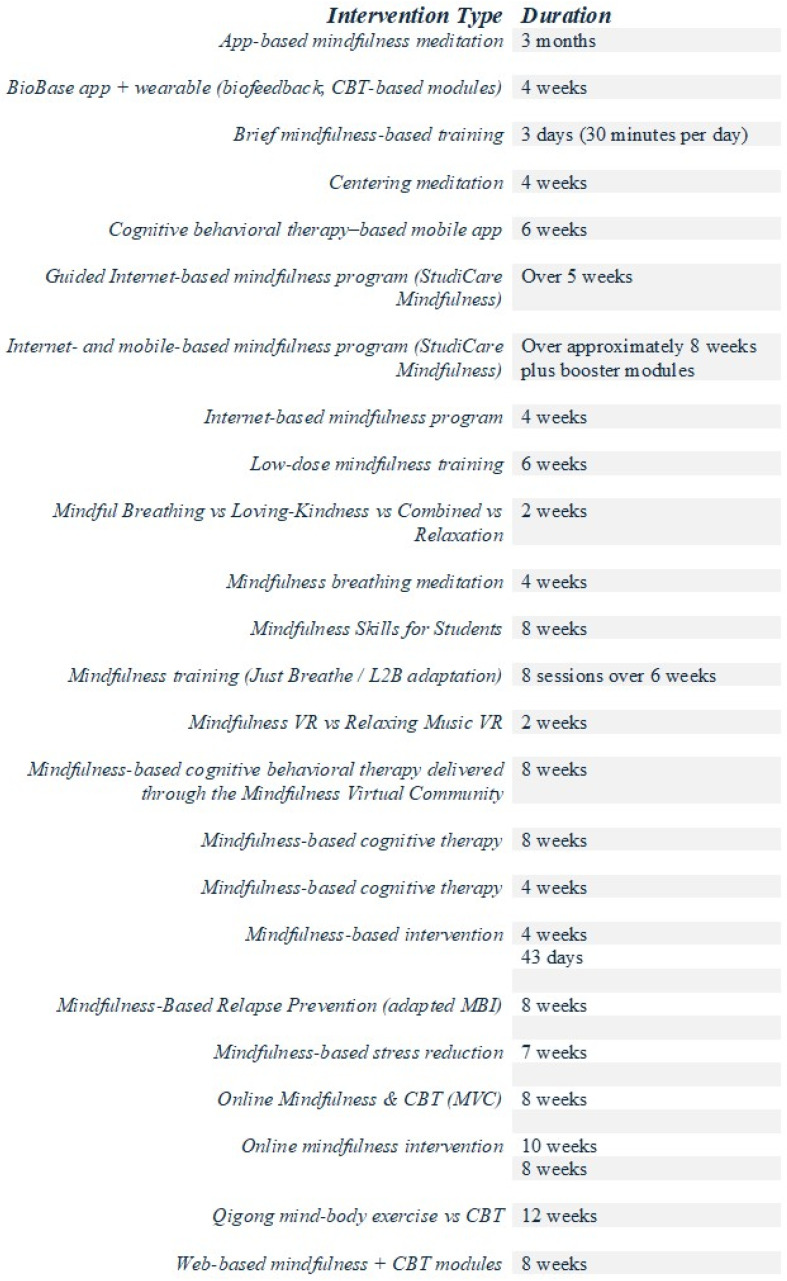
Types of mindfulness-based interventions across included studies.

**Figure 6:**
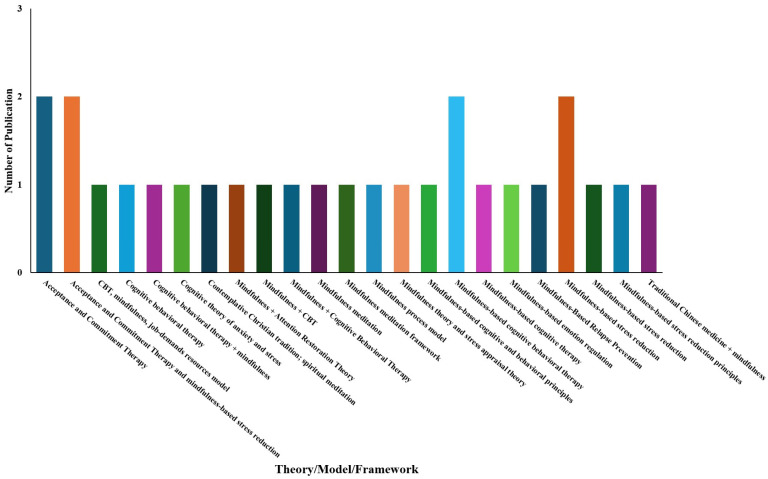
Theoretical frameworks and models underlying the included interventions.

The theoretical frameworks and intervention models used across the included studies are summarized in Table 6. Mindfulness-based interventions were grounded in diverse theoretical approaches, primarily integrating mindfulness principles with cognitive and behavioral frameworks. The distribution of frameworks and intervention are presented in Figure 5 and Figure 6.

This figure summarizes the diversity of intervention types, including app-based mindfulness, cognitive behavioral therapy–integrated programs, breathing meditation, and hybrid mindfulness approaches.

This figure illustrates the range of theoretical models used, including mindfulness-based cognitive therapy (MBCT), acceptance and commitment therapy (ACT), cognitive behavioral therapy (CBT), and integrative mindfulness frameworks.

#### 3.10.1. Dominant Theoretical Approaches

A substantial proportion of studies used mindfulness-based cognitive-behavioral therapy (MB-CBT) or closely related models. For example, El Morr et al. (2020) [28], Ahmad et al. (2020) [1], and Ritvo et al. (2021) [35] implemented MB-CBT-based interventions combining mindfulness practices with cognitive restructuring and peer-supported environments. Similarly, Liu et al. (2024) [26] and Rodriguez et al. (2021) [36] applied mindfulness-based cognitive therapy (MBCT). Several studies also incorporated Acceptance and Commitment Therapy (ACT) alongside mindfulness-based stress reduction (MBSR). Küchler et al. (2022, 2023) [16,25] and Balci et al. (2023, 2024) [4,17] used ACT-informed approaches.

#### 3.10.2. Mindfulness-Based Stress Reduction and Meditation Frameworks

Many studies were based on MBSR and mindfulness meditation frameworks. Karing and Beelmann (2021) [18], Komariah et al. (2023) [38], Barcaccia et al. (2024) [39], and Gao et al. (2024) [20] applied MBSR approaches. Similarly, Flett et al. (2020) [30] and de Sousa et al. (2021) [34] used meditation-based interventions, while Bolognino et al. (2023) [37] applied the mindfulness process model.

#### 3.10.3. Cognitive and Behavioral Frameworks

Several studies incorporated CBT or CBT-informed models. McCloud et al. (2020) [32] implemented a CBT-based digital intervention. Ponzo et al. (2020) [33] combined CBT with mindfulness. Ellison et al. (2024) [15] applied cognitive theory of anxiety and stress, while Ritvo et al. (2021) [35] used stress appraisal theory.

#### 3.10.4. Alternative and Integrative Frameworks

Some studies used integrative or non-traditional frameworks. Lu et al. (2020) [31] combined traditional Chinese medicine with mindfulness, while Dorais and Gutierrez (2021) [29] applied a contemplative spiritual framework. Zheng et al. (2024) [40] integrated mindfulness with Attention Restoration Theory using virtual reality, and Bai et al. (2020) [27] focused on mindfulness-based emotion regulation.

#### 3.10.5. Digital and Technology-Enhanced Frameworks

Digital and technology-enhanced delivery was common. Interventions included mobile applications, web-based platforms, and virtual communities. For example, El Morr et al. (2020) [28] and Ritvo et al. (2021) [35] used the Mindfulness Virtual Community, while Gao et al. (2024) [20] and Ponzo et al. (2020) [33] incorporated app-based and wearable technologies.

### 3.11. Comparative Synthesis of In-Person and Digital Mindfulness Interventions

Table 7 presents the comparison of in-person and digital interventions. Digital interventions constituted the majority and were delivered through mobile, web-based, and internet platforms. In-person interventions were fewer and typically involved structured, instructor-led MBCT programs. Digital interventions reported improvements in depression, anxiety, and mindfulness outcomes, with variable findings for stress. In-person interventions also demonstrated positive effects, particularly for stress reduction. Engagement and adherence differed by delivery mode. In-person interventions reported higher attendance, whereas digital interventions showed variability in adherence. Follow-up findings indicated more sustained effects for in-person interventions, while digital outcomes depended on continued engagement.

### 3.12. Methodological Quality Assessment

The methodological quality of the included randomized controlled trials (*n* = 24) was assessed using the Joanna Briggs Institute (JBI) critical appraisal checklist (Table 8). Overall, the included studies demonstrated low to moderate risk of bias. All studies reported random allocation of participants, and most demonstrated baseline comparability between intervention and control groups. Outcome measurement was consistent and reliable across studies, with validated instruments commonly used to assess stress, anxiety, and depression outcomes. Appropriate statistical analyses were also applied.

However, several methodological limitations were observed. Participant blinding and intervention provider blinding were largely infeasible due to the behavioral nature of mindfulness-based interventions. Allocation concealment was unclear in a substantial proportion of studies. In some trials, incomplete follow-up or attrition was reported, particularly in digitally delivered interventions. Additionally, analysis by assigned groups was not consistently reported across all studies. Despite these limitations, the majority of studies satisfied key JBI criteria. Based on the predefined JBI quality classification criteria, the majority of included studies were rated as high quality, while the remaining studies were classified as moderate quality. No studies were excluded on the basis of methodological quality.

## 4. Discussion

### 4.1. Summary of Key Findings

This review synthesized evidence from randomized controlled trials examining mindfulness-based interventions among university students, integrating findings related to study characteristics, intervention designs, theoretical frameworks, and mental health outcomes (Table 3, Table 4, Table 5 and Table 6). Overall, the findings indicate that mindfulness-based interventions are associated with meaningful improvements in mental health outcomes, with the most consistent effects observed for reductions in depression and anxiety. In contrast, findings related to stress outcomes were more heterogeneous. The included studies demonstrated substantial variability in intervention design, duration, theoretical frameworks, and delivery modes, including in-person, digital, and hybrid formats. Despite this heterogeneity, the overall pattern of results suggests that mindfulness-based interventions are adaptable, scalable, and effective across diverse student populations [16,32,36,42].

These findings highlight that mindfulness-based interventions may be particularly effective in targeting internalizing symptoms such as depression and anxiety, while their impact on stress may depend on contextual and individual-level factors such as engagement, intervention intensity, and measurement approaches. The temporal distribution of studies further suggests an increase in research interest in mindfulness-based interventions over recent years (Figure 2). All these findings are shown in Table 3, Table 4, Table 5 and Table 6 and Figure 2, Figure 3 and Figure 4.

### 4.2. Effectiveness of Mindfulness-Based Interventions

#### 4.2.1. Depression Outcomes

The most robust and consistent effects were observed for depressive symptoms. Multiple studies reported statistically significant reductions in depression, particularly those employing mindfulness-based cognitive therapy and acceptance-based approaches [16,32,36,42]. These findings are consistent with the Section 3, which demonstrated that interventions targeting maladaptive cognitive processes, such as rumination and negative automatic thoughts, produced meaningful improvements in depressive symptoms [5,29]. The effectiveness of mindfulness-based interventions for depression may be explained by their ability to enhance present-moment awareness, promote cognitive decentering, and reduce repetitive negative thinking. These mechanisms directly address core features of depressive symptomatology, including rumination and cognitive rigidity. These findings align with previous systematic reviews and meta-analyses demonstrating the effectiveness of mindfulness-based interventions for depression across both clinical and non-clinical populations. However, variability remains, as some studies [37] reported non-significant effects, suggesting that contextual factors such as adherence, intervention format, and participant characteristics may influence outcomes. These findings correspond with Table 5.

#### 4.2.2. Anxiety Outcomes

Mindfulness-based interventions also demonstrated generally positive effects on anxiety, although findings were less consistent than those for depression. Several studies reported significant reductions in anxiety symptoms, particularly interventions integrating mindfulness with cognitive-behavioral strategies [24,38,40]. Additionally, technology-enhanced interventions, including app-based and hybrid models, were associated with improvements in anxiety outcomes [28,31]. Nevertheless, some studies reported non-significant or short-term effects. For example, some studies [34] found no significant reduction in anxiety, while others [42] observed improvements that were not sustained over time. Additional findings [35] indicated that baseline mindfulness levels may moderate intervention effectiveness. These findings suggest that mindfulness-based interventions may influence anxiety through mechanisms such as improved attentional control, reduced hypervigilance, and enhanced emotional regulation. However, variability in outcomes indicates that effectiveness may depend on intervention intensity, engagement, and individual differences. These results are reflected in Table 5.

#### 4.2.3. Stress Outcomes

In contrast to depression and anxiety, findings for stress outcomes were more heterogeneous. While several studies reported significant reductions in perceived stress [31,34,38], others reported no significant changes [4,17,42]. This variability may be attributed to differences in intervention duration, adherence, delivery formats, and measurement approaches. Additionally, stress was conceptualized inconsistently across studies, with some measuring perceived stress directly and others assessing related constructs such as rumination or sleep quality. Importantly, several studies demonstrated indirect or preventive effects. For example, some studies [43] reported stabilization of stress-related emotional responses, others [5] observed reductions in rumination, and others [36] reported improvements in sleep quality. These findings suggest that mindfulness-based interventions may enhance stress resilience and coping capacity rather than consistently reducing perceived stress levels. These heterogeneous patterns are summarized in Table 5.

### 4.3. Role of Intervention Design and Delivery

The included studies demonstrated substantial heterogeneity in intervention design, delivery modes, and intensity. Interventions ranged from traditional in-person group sessions to fully digital platforms, including mobile applications, web-based modules, and virtual reality environments [41,42]. Despite this variability, positive outcomes were observed across multiple formats, suggesting that mindfulness-based interventions are adaptable and scalable. However, interventions that incorporated structured guidance, instructor support, or interactive components tended to produce more consistent outcomes than fully self-guided programs [1,14]. These findings indicate that while digital interventions enhance accessibility, the inclusion of human support may be critical for optimizing engagement and effectiveness. These variations are illustrated in Table 4, Figure 3 and Figure 5.

### 4.4. Theoretical Implications

This review highlights that mindfulness-based interventions are predominantly grounded in integrative theoretical frameworks combining mindfulness principles with cognitive and behavioral models, including CBT, MBCT, and ACT. These frameworks provide a multidimensional approach targeting attentional regulation, cognitive restructuring, and emotional processing [32,36,42]. The findings suggest that hybrid models integrating mindfulness with structured cognitive strategies may be particularly effective for depression and anxiety outcomes. From a theoretical perspective, mindfulness functions both as an attentional training mechanism and a cognitive–affective regulatory process, enabling individuals to disengage from maladaptive thought patterns such as rumination and worry. The incorporation of ACT further emphasizes psychological flexibility and values-based action, which may contribute to sustained behavioral change. Additionally, alternative frameworks, including emotion regulation models and Attention Restoration Theory, expand the conceptual understanding of mindfulness-based interventions [33,34]. These theoretical distributions are summarized in Table 6 and Figure 6.

### 4.5. Digital and Technology-Enhanced Frameworks

A notable trend across studies was the integration of mindfulness frameworks into digital and technology-enhanced platforms (Table 6). Interventions delivered through mobile applications, online platforms, and virtual communities were grounded in established theoretical models while enhancing accessibility and scalability. For example, El Morr et al. (2020) [28] and Ritvo et al. (2021) [35] utilized the Mindfulness Virtual Community, while Gao et al. (2024) [20] and Ponzo et al. (2020) [33] incorporated app-based and wearable technologies. Overall, mindfulness-based interventions for university students were supported by diverse theoretical frameworks, including mindfulness-based stress reduction, cognitive behavioral therapy, and acceptance and commitment therapy. The findings indicate a convergence toward hybrid approaches that integrate mindfulness with cognitive, behavioral, and technological components, reflecting both the flexibility and adaptability of these interventions across delivery platforms and populations.

### 4.6. Comparison of Delivery Modes: Digital vs. In-Person Interventions

The comparative synthesis highlights key differences between digital and in-person mindfulness interventions among university students (Table 7). Digital interventions demonstrated broad effectiveness across depression, anxiety, and mindfulness outcomes and offered advantages in accessibility, scalability, and flexibility. However, engagement and adherence remain critical challenges, with several studies reporting variability in participation and completion rates [4,16,35]. In contrast, in-person interventions involved more structured, instructor-led formats and demonstrated stronger adherence and potentially more sustained benefits. Instructor guidance, scheduled sessions, and interpersonal interaction may enhance accountability and continuity of practice. These findings suggest that digital and in-person interventions should be viewed as complementary rather than competing approaches. While digital interventions provide scalable access to mental health support, in-person interventions may facilitate deeper engagement and longer-term behavioral change. Hybrid models that integrate digital accessibility with structured guidance may represent an optimal strategy for maximizing both reach and effectiveness.

### 4.7. Methodological Quality and Risk of Bias

Overall, the included randomized controlled trials (*n* = 24) demonstrated low to moderate risk of bias, indicating generally acceptable methodological quality. Most studies reported adequate randomization, baseline comparability, use of validated outcome measures, and appropriate statistical analyses, supporting the credibility of the findings. However, several limitations were noted. Blinding of participants and intervention providers was largely not feasible due to the behavioral nature of mindfulness-based interventions, potentially introducing performance and expectancy bias. Allocation concealment was frequently unclear, and some studies reported incomplete follow-up or attrition, particularly in digitally delivered interventions, which may affect the precision of effect estimates. Despite these limitations, the overall consistency in study design and outcome measurement strengthens confidence in the evidence. Nevertheless, findings should be interpreted with caution, and future research should prioritize clearer reporting of allocation procedures, improved retention strategies, and the inclusion of more objective outcome measures. This section is supported by Table 8.

### 4.8. Limitations

This review has several limitations. First, substantial heterogeneity in intervention design, delivery modes, and outcome measures limits comparability across studies. Second, reliance on self-reported measures may introduce reporting bias. Third, variability in adherence and engagement, particularly in digital interventions, may influence observed outcomes. Fourth, a formal certainty-of-evidence assessment using the GRADE approach was not conducted. Given the substantial heterogeneity in intervention characteristics, delivery modalities, outcome measures, and follow-up periods, the review focused on methodological quality assessment using the JBI critical appraisal tool. Future systematic reviews and meta-analyses may benefit from incorporating GRADE assessments to evaluate overall certainty of evidence across outcomes. Finally, limited long-term follow-up restricts the ability to assess the sustainability of intervention effects.

### 4.9. Practical Implications

The findings of this review have important implications for university-based mental health services. The consistent evidence supporting reductions in depression and anxiety indicates that mindfulness-based interventions can serve as effective, non-pharmacological strategies for addressing common mental health challenges among students. This aligns with broader evidence from mind–body interventions, such as Kundalini Yoga, which has demonstrated clinical effectiveness across multiple physical and mental health outcomes in randomized controlled trials [44]. Digital and technology-based delivery platforms, including mobile applications and web-based modules, enhance accessibility and scalability, allowing flexible engagement. However, interventions incorporating guided support or structured engagement appear to produce more consistent outcomes than fully self-guided programs. Universities should consider integrating mindfulness-based interventions into student health services, academic curricula, and wellness programs.

### 4.10. Public Health Implications

The findings of this review highlight the broader public health relevance of mindfulness-based interventions for addressing mental health challenges among university students. These interventions represent scalable and cost-effective strategies that can support prevention, early intervention, and self-management of stress, anxiety, and depression. The expansion of digital delivery formats enhances reach and accessibility, particularly for students facing barriers to traditional in-person services, including stigma, time constraints, and limited access to care. From a policy perspective, the evidence supports integrating mindfulness-based interventions into institutional health promotion initiatives and national mental health strategies targeting young adults. Such approaches may contribute to reducing disparities in access to mental health services and strengthening population-level mental health outcomes.

### 4.11. Future Research Directions

Future research should address the methodological and conceptual gaps identified in this review. Greater standardization of outcome measures and intervention protocols is needed to improve comparability across studies and facilitate meta-analytic synthesis, particularly through the use of validated and consistent instruments for stress, anxiety, and depression. Longitudinal studies with extended follow-up periods are essential to assess the sustainability of intervention effects. In addition, future studies should examine moderators and mediators of effectiveness, including baseline mental health status, engagement levels, personality traits, and delivery format, to support more targeted and personalized interventions. Finally, emerging technology-enhanced approaches, such as virtual reality and wearable-integrated systems, warrant further investigation to evaluate their effectiveness, feasibility, and potential to enhance engagement in diverse student populations.

## 5. Conclusions

In conclusion, this review provides comprehensive evidence that mindfulness- and meditation-based interventions may offer beneficial effects and represent adaptable approaches for supporting mental health among university students. The available evidence suggests that these interventions are associated with improvements in depression and anxiety outcomes, while effects on stress appear more variable and may reflect improvements in stress resilience, coping capacity, and stress management abilities rather than direct symptom reduction. Although the findings indicate potential utility for university mental health services, the substantial heterogeneity in intervention characteristics, delivery modalities, outcome measures, and follow-up periods warrants cautious interpretation of the evidence. The increasing use of digital and technology-enhanced delivery platforms further underscores the potential for scalable and flexible interventions that can reach diverse student populations. The integration of mindfulness with cognitive and behavioral frameworks, together with advances in digital delivery, suggests that these interventions may represent a valuable component of comprehensive student mental health strategies. Continued research is needed to optimize intervention design, improve long-term effectiveness, evaluate comparative effectiveness across delivery modalities, and expand implementation across diverse educational and cultural settings.

## Figures and Tables

**Table 1:** PICOS Framework and Inclusion Criteria.

PICOS Element	Inclusion Criteria
Population (P)	University or college students aged ≥ 18 years enrolled in undergraduate or graduate programs.
Intervention (I)	Any mindfulness- or meditation-based intervention, including Mindfulness-Based Stress Reduction (MBSR), Mindfulness-Based Cognitive Therapy (MBCT), breathing meditation, yoga-based meditation, mindful awareness practices, compassion-focused meditation, digital mindfulness applications, web-based mindfulness or mindfulness–CBT hybrid programs, and peer-supported meditation interventions.
Comparator (C)	Waitlist control, no-treatment control, usual care, attention control, psychoeducation, or alternative non-mindfulness interventions.
Outcomes (O)	Validated measures of stress, anxiety, and/or depression assessed using standardized instruments such as the Perceived Stress Scale (PSS), Depression Anxiety Stress Scales (DASS-21 or DASS-42), Generalized Anxiety Disorder Scale (GAD-7), Beck Anxiety Inventory (BAI), State–Trait Anxiety Inventory (STAI), Patient Health Questionnaire (PHQ-8 or PHQ-9), or Hospital Anxiety and Depression Scale (HADS-A/HADS-D).
Study Design (S)	Randomized controlled trials (RCTs)
Other Criteria	Studies published between 2020 and 2025, written in English, and published in peer-reviewed journals.

**Table 2:** Search strategies used in each database.

Database	Search Query
PubMed/MEDLINE	(“mindfulness”[MeSH] OR “meditation”[MeSH] OR “mindfulness-based” OR MBSR OR MBCT OR “breathing meditation” OR “mindful breathing” OR “body scan”) AND (“students”[MeSH] OR “university students” OR “college students” OR undergraduate OR graduate) AND (“stress, psychological”[MeSH] OR stress OR anxiety OR depression OR “mental health”) AND (“randomized controlled trial”[Publication Type] OR randomized OR randomised OR RCT). Filters: English; 2020–2025.
PsycINFO (APA Thesaurus)	(DE “Mindfulness” OR mindfulness OR meditation OR “mindfulness-based” OR MBSR OR MBCT) AND (DE “College Students” OR “university student” OR undergraduate OR graduate) AND (DE “Psychological Stress” OR stress OR anxiety OR depression) AND (DE “Randomized Controlled Trials” OR randomized OR controlled). Limiters: English; 2020–2025; Peer-reviewed.
CINAHL (EBSCO)	(MH “Mindfulness” OR MH “Meditation” OR mindfulness OR meditation OR MBSR OR MBCT) AND (MH “Students, College” OR “university students” OR “college students”) AND (MH “Stress, Psychological” OR stress OR anxiety OR depression) AND (MH “Randomized Controlled Trials” OR randomized OR controlled). Filters: English; 2020–2025.
Scopus	(TITLE-ABS-KEY (mindfulness OR meditation OR “mindfulness-based” OR “mindful breathing” OR “meditation app”) AND TITLE-ABS-KEY (“university student” OR “college student”) AND TITLE-ABS-KEY (stress OR anxiety OR depression) AND TITLE-ABS-KEY (randomized OR “controlled trial”)) AND (LIMIT-TO (LANGUAGE, “English”)) AND (PUBYEAR > 2019 AND PUBYEAR < 2026).
Web of Science	TS = (mindfulness OR meditation OR “mindfulness-based” OR MBSR OR MBCT) AND TS = (“university students” OR “college students”) AND TS = (stress OR anxiety OR depression) AND TS = (randomized OR controlled). Refined by: Document type = Article; Language = English; Years = 2020–2025.

**Table 3:** Characteristics of Included studies (*n* = 24).

Author (Year)	Country	Sample Size	Population	Intervention Type	Delivery Mode	Duration
Ahmad et al. (2020) [1]	Canada	113	Undergraduate students	Web-based mindfulness & CBT modules	Online modules, forums & videoconference	8 weeks
Bai et al. (2020) [27]	USA	52	College students	Mindfulness training (Just Breathe/L2B adaptation)	In-person group sessions	8 sessions over 6 weeks
El Morr et al. (2020) [28]	Canada	160	Undergraduate university students	Mindfulness-based cognitive behavioral therapy delivered through the Mindfulness Virtual Community	Online videos, anonymous discussion boards, and group videoconferencing	8 weeks
Dorais and Gutierrez (2021) [29]	USA	150	College students	Centering meditation	Online, self-guided twice daily	4 weeks
Flett et al. (2020) [30]	New Zealand	250	University students	App-based mindfulness meditation	Mobile application	3 months
Galante et al. (2020) [14]	United Kingdom	616	University students	Mindfulness Skills for Students	In-person group sessions	8 weeks
Lu et al. (2020) [31]	China	95	College students	Qigong mind–body exercise vs CBT	In-person sessions	12 weeks
McCloud et al. (2020) [32]	United Kingdom	168	University students	Cognitive behavioral therapy-based mobile app	Smartphone app	6 weeks
Ponzo et al. (2020) [33]	United Kingdom	123	University students	BioBase app & wearable (biofeedback, CBT-based modules)	Mobile app & wearable	4 weeks
de Sousa et al. (2021) [34]	Brazil	40	Undergraduate and graduate students	Brief mindfulness-based training	In-person audio-guided meditation sessions	3 days (30 min per day)
Karing and Beelmann (2021) [18]	Germany	71	University students	Low-dose mindfulness training	In-person group sessions	6 weeks
Ritvo et al. (2021) [35]	Canada	154	Undergraduate university students	Mindfulness-based cognitive behavioral therapy delivered through the Mindfulness Virtual Community	Online videos, anonymous discussion boards, and group videoconferencing	8 weeks
Rodriguez et al. (2021) [36]	China	54	University students	Internet-based mindfulness program	Web-based course	4 weeks
Küchler et al. (2022) [25]	Germany, Austria, Switzerland	150	College students	Guided Internet-based mindfulness program (StudiCare Mindfulness)	Online modules with weekly e-coach feedback	5 modules over 5 weeks
Küchler et al. (2023) [16]	Germany, Austria, Switzerland	387	College students	Internet- and mobile-based mindfulness program (StudiCare Mindfulness)	Online modules with optional guidance on demand	7 modules over approximately 8 weeks plus booster modules
Balci et al. (2023) [4]	Germany	40	International university students	Online mindfulness intervention	Web-based modules	8 weeks
Bolognino et al. (2023) [37]	United States	52	University students	Mindful Breathing vs Loving-Kindness vs Combined vs Relaxation	Audio-guided self-practice	2 weeks
Gallo et al. (2023) [19]	Brazil	136	University students	Mindfulness-Based Relapse Prevention (adapted MBI)	In-person group sessions	8 weeks
Komariah et al. (2023) [38]	Indonesia	122	University students	Mindfulness breathing meditation	Online (Zoom & self-practice)	4 weeks
Balci et al. (2024) [17]	Turkey	58	Turkish-speaking university students	Online mindfulness intervention	Web-based modules	10 weeks
Barcaccia et al. (2024) [39]	Italy and New Zealand	486	University students	Mindfulness-based intervention	Online & in-class	4 weeks
Ellison et al. (2024) [15]	United States	289	College students	Mindfulness-based cognitive therapy	Smartphone application	4 weeks
Liu et al. (2024) [26]	China	56	Undergraduate students	Mindfulness-based cognitive therapy	In-person	8 weeks
Zheng et al. (2024) [40]	China	60	University students	Mindfulness VR vs Relaxing Music VR	Virtual reality headset	2 weeks

**Table 4:** Intervention Characteristics and Delivery Features of Included Mindfulness-Based Programs (*n* = 24).

Author (Year)	Objectives	Intervention Model	Core Components	Session Format	Frequency	Instructor Support	Delivery Platform
Ahmad et al. (2020) [1]	Reduce depression, anxiety, and stress	Mindfulness & CBT	Psychoeducation, mindfulness practice, peer forums, guided videoconferences	Modules & group video	3 modules/week & optional sessions	A mental health professional moderated	Web-based MVC platform
Bai et al. (2020) [27]	Improve daily stress response & emotion regulation	Learning 2 Breathe (college adaptation)	Body awareness, thought awareness, emotion awareness, integration, and reducing self-judgment	Group sessions	2 times per week for weeks 1 to 2, then 1 time per week	Facilitators led sessions & audio practices	In-person
El Morr et al. (2020) [28]	Reduce depression, anxiety, stress. & increase mindfulness	Mindfulness-based cognitive behavioral therapy	Educational videos, mindfulness practice videos, peer discussion boards, and group videoconferencing	Self-paced video modules and 20 min group videoconferences	Weekly module release and videoconferences biweekly	Counselor trained in mindfulness	Web-based Mindfulness Virtual Community
Dorais and Gutierrez (2021) [29]	Reduce distress and improve adjustment	App-based mindfulness	Guided meditations, breathing, body scan	Self-guided app sessions	At discretion	None	Headspace app (Mobile application)
Flett et al. (2020) [30]	Improve resilience and reduce distress	Mindfulness Skills for Students	Meditation, inquiry, reflection	Group sessions	Weekly	Certified mindfulness teacher	In-person
Galante et al. (2020) [14]	Reduce PSU, anxiety, loneliness	Mind–Body Exercise (Qigong) vs CBT	Qigong movement, breathing & CBT relapse prevention	90 min sessions	2 times per week for 12 weeks	Certified therapists	In-person
Lu et al. (2020) [31]	Reduce anxiety and depression	Cognitive behavioral therapy–based digital program	Relaxation, mood tracking, and thought challenging	Self-guided	Weekly minimum	None	Smartphone app
McCloud et al. (2020) [32]	Reduce anxiety, stress, and depression	Biofeedback & CBT & mindfulness	Psychoeducation, breathing, EMA mood tracking, wearable data	Self-guided modules	Daily use encouraged	Minimal (technical only)	Mobile app & wearable
Ponzo et al. (2020) [33]	Reduce stress & increase mindfulness	Centering meditation	Sacred word focus, attention redirection, twice-daily practice	Self-guided	Two times per day.	Minimal (online instructions only)	Online
de Sousa et al. (2021) [34]	Reduce anxiety, stress, and negative affect, increase state mindfulness	Mindfulness meditation	Audio-guided meditation focusing on breath and body sensations	In-person guided meditation	30 min per day for 3 days	Research staff present during sessions	Laboratory setting
Karing and Beelmann (2021) [18]	Improve mindfulness and self-efficacy	Low-dose mindfulness training	Breathing, body scan, mindful eating, yoga	Group sessions	Weekly	Instructor-led	In-person
Ritvo et al. (2021) [35]	Reduce depression, anxiety, and perceived stress; improve mindfulness	Mindfulness-based cognitive behavioral therapy	Educational videos, mindfulness practice videos, peer discussion boards, and group videoconferencing	Self-paced video modules and 20 min group videoconferences	Weekly module release; videoconferences biweekly	Moderator with a master’s degree in psychology	Web-based Mindfulness Virtual Community
Rodriguez et al. (2021) [36]	Improve engagement and mental health	Internet-based mindfulness	Videos, audio practices, homework	Online modules	Weekly	Peer counselor support	Web platform
Küchler et al. (2022) [25]	Improve mindfulness and reduce stress, depression, and anxiety	Acceptance and Commitment Therapy and Mindfulness-Based Stress Reduction	Psychoeducation, mindfulness exercises, meditation audios, values, and goals work	Online modules	Weekly modules	Weekly personalized feedback from e-coach	StudiCare digital platform
Küchler et al. (2023) [16]	Improve mindfulness and mental health outcomes	Acceptance and Commitment Therapy and Mindfulness-Based Stress Reduction	Psychoeducation, mindfulness exercises, self-reflection tasks, goal-setting, meditation audios	Online modules	Weekly modules; optional booster modules	Guidance on demand (optional)	StudiCare digital platform
Balci et al. (2023) [4]	Improve mindfulness and well-being	Online mindfulness program	Psychoeducation, meditation, journaling	Web modules	Weekly	E-coach feedback	Web platform
Bolognino et al. (2023) [37]	Compare MBM vs. LKM	Mindful Breathing & Loving-Kindness	Breath awareness, compassion meditation	10 min guided audio	Daily for 2 weeks	None (self-guided)	Audio recordings
Gallo et al. (2023) [19]	Reduce stress, depression, and insomnia	Mindfulness-Based Relapse Prevention (adapted)	Awareness, acceptance, reactivity management, home practice	Weekly 1.5 h group sessions	8 sessions (Weekly)	Certified mindfulness instructors	In-person
Komariah et al. (2023) [38]	Reduce depression, anxiety, and stress	Mindfulness breathing meditation	Breath awareness, present-moment attention	Guided Zoom & self-practice	Daily	Research assistants guided the first 2 weeks	Online (Zoom & WhatsApp)
Balci et al. (2024) [17]	Improve mindfulness in Turkish students	Online mindfulness program	Meditation, values work, stress coping.	Web modules	Weekly	E-coach feedback	Web platform
Barcaccia et al. (2024) [39]	Reduce depression, rumination, and anxiety	Mindfulness-based stress reduction	Breathing meditation, guided sitting practice	In-class & daily practice	weekly and daily	Licensed psychologist	Online & classroom
Ellison et al. (2024) [15]	Reduce anxiety and stress	Mindfulness-based cognitive therapy	Meditation, cognitive restructuring	App-based sessions	Daily practice encouraged	None	Smartphone application
Liu et al. (2024) [36]	Treat major depressive disorder	Mindfulness-based cognitive therapy	Meditation, cognitive restructuring	Group sessions	Weekly	MBCT-trained clinician	In-person
Zheng et al. (2024) [40]	Reduce anxiety, stress, and depression	Mindfulness VR vs. Relaxing Music VR	Guided mindfulness, immersive VR nature scenes	Individual VR sessions	Alternate days for 2 weeks	Research staff supervision	VR headset

**Table 5:** Characteristics and Mental Health Outcomes (Stress, Anxiety, and Depression) of Included Studies (*n* = 24).

Author (Year)	Outcome Measurement Instruments	Stress Outcome (Effect Size/Statistic, *p*-Value, Interpretation)	Anxiety Outcome (Effect Size/Statistic, *p*-Value, Interpretation)	Depression Outcome (Effect Size/Statistic, *p*-Value, Interpretation)
Ahmad et al. (2020) [1]	PHQ-9; BAI; PSS; QoL	Significant reduction (*p* < 0.001), indicating great improvement in stress levels	Partial improvement (*p* = 0.01), indicating modest improvement in anxiety	Significant reduction (*p* < 0.001), indicating great improvement in depression
Bai et al. (2020) [27]	EMA; stress; emotion regulation measures	No statistically significant reduction: intervention prevented worsening of stress-related responses, indicating a stabilization effect	Anxiety was not directly assessed, as the study focused on momentary stress and emotion regulation	Depression was not directly assessed, as clinical symptom outcomes were not evaluated
El Morr et al. (2020) [28]	PHQ-9; BAI; PSS; FFMQ-SF	No significant change (B = 0.64 & *p* = 0.48), indicating no meaningful change in stress levels	Significant reduction (B = −4.82 & *p* = 0.006), indicating improvement in anxiety symptoms	Significant reduction (B = −2.21 & *p* = 0.01), indicating improvement in depressive symptoms
Dorais and Gutierrez (2021) [29]	Distress; resilience; self-efficacy scales	Small improvement (R^2^ = 0.12), indicating a weak effect on stress-related outcomes	No statistically significant improvement, indicating a weak effect	No statistically significant improvement, indicating a weak effect
Flett et al. (2020) [30]	CORE; WEMWBS	Psychological distress reduced (β = −0.22 & *p* < 0.001), indicating improvement in stress-related outcomes	Anxiety is not separately measured, as outcomes are focused on general distress	Depression is not separately measured, as outcomes are focused on general distress
Galante et al. (2020) [14]	Anxiety & stress scales	Significant reduction (*p* < 0.001), indicating great improvement in stress levels	Significant reduction (*p* < 0.001), indicating great improvement in anxiety symptoms	Improvement reported as a secondary outcome
Lu et al. (2020) [31]	HADS	Stress was not directly assessed in this study	Significant reduction at week 4 (MD = −1.94 & *p* = 0.001), but not sustained at week 6	Significant reduction at week 6 (MD = −1.56 & *p* = 0.006), indicating delayed improvement
McCloud et al. (2020) [32]	DASS-21	Significant reduction (*p* ≤ 0.05), indicating improvement in stress levels (moderate to large effect size)	Significant reduction (*p* ≤ 0.05), indicating improvement in anxiety symptoms (Cohen’s d = 0.67–0.81)	Significant reduction (*p* ≤ 0.05), indicating improvement in depressive symptoms (large effect, d ≈ 0.99)
Ponzo et al. (2020) [33]	PSS & CAMS-R	Significant reduction (*p* < 0.05), indicating improvement in stress levels	Anxiety was not directly assessed	Depression was not directly assessed
de Sousa et al. (2021) [34]	PSS, STAI, PANAS & cortisol	Significant reduction (*p* < 0.05), mediated by increased mindfulness, indicating meaningful improvement in stress levels	Significant reduction in anxiety state (*p* < 0.05), particularly in the high trait mindfulness subgroup	Depression was not directly assessed
Karing and Beelmann (2021) [18]	Mental health scales	Significant improvement (d = 0.43–1.06), indicating moderate-to-large effects, though stress not isolated	Mixed findings; not consistently statistically significant	No statistically significant improvement in depression
Ritvo et al. (2021) [35]	PHQ-9, BAI, PSS & FFMQ-SF	Significant reduction (B = −2.31; *p* = 0.03), indicating improvement in stress levels	No statistically significant improvement (*p* = 0.31)	No statistically significant improvement (*p* = 0.64)
Rodriguez et al. (2021) [36]	Self-report scales	Large reduction (d = 1.13; *p* < 0.05), indicating strong improvement in stress levels	Large reduction (d = 0.89; *p* < 0.05), indicating strong improvement in anxiety	Large reduction (d = 0.95; *p* < 0.05), indicating strong improvement in depression
Küchler et al. (2022) [25]	FMI, PHQ-9, GAD-7 & PSQ-20	Large reduction (Cohen’s d = −0.92), indicating a substantial improvement in stress levels	Moderate reduction (Cohen’s d = −0.50), indicating a meaningful improvement in anxiety symptoms	Large reduction (Cohen’s d = −0.87), indicating a substantial improvement in depressive symptoms
Küchler et al. (2023) [16]	PHQ-9, GAD-7, PSS & mindfulness scales	Significant reduction (d about −0.92), indicating moderate-to-large improvement	Significant reduction (d about −0.50), indicating moderate improvement	Significant reduction (d ≈ −0.87), indicating a large improvement
Balci et al. (2023) [4]	Mindfulness; stress; anxiety; depression; wellbeing scales	No statistically significant improvement, indicating no meaningful change in stress levels	Significant reduction (β = −0.42; *p* < 0.05), indicating improvement in anxiety	No statistically significant improvement in depression
Bolognino et al. (2023) [37]	Mental health, depression; process variables	Not specifically reported	Not clearly reported	Significant reduction in depression (*p* < 0.001 & η^2^ = 0.447, large effect), indicating improvement over time
Gallo et al. (2023) [19]	PSS-10; PHQ-9; STAI	Significant reduction (*p* < 0.001), interpretation depends on coding direction	No statistically significant improvement	Significant reduction (*p* < 0.01), indicating improvement in depression
Komariah et al. (2023) [38]	DASS-42	Significant reduction (*p* = 0.007), indicating improvement in stress	Significant reduction (*p* = 0.042), indicating improvement in anxiety	No statistically significant improvement
Balci et al. (2024) [17]	PSS-4; GAD-7; PHQ-9; WHO-5	No statistically significant effect on perceived stress (β = 0.09, 95% CI: −0.47 to 0.64, *p* = 0.75), indicating no meaningful improvement	No statistically significant effect on anxiety symptoms (β = −0.23, 95% CI: −0.79 to 0.33, *p* = 0.41), indicating no significant reduction	No statistically significant effect on depressive symptoms (β = 0.13, 95% CI: −0.36 to 0.63, *p* = 0.66), indicating no meaningful change
Barcaccia et al. (2024) [39]	BDI; RRS; STAI	Stress not directly measured; reduction inferred via decreased rumination	Significant reduction (*p* < 0.001), indicating great improvement in anxiety	Significant reduction (*p* < 0.001), indicating great improvement in depression
Ellison et al. (2024) [15]	Perceived Stress Scale (PSS-4); State-Trait Anxiety Inventory (STAI)	Moderate reduction in chronic stress following MBCT intervention (Cohen’s d_rm = 0.47, 95% CI: 0.33–0.61; *p* < 0.001), indicating a significant and meaningful improvement in perceived stress levels	Small-to-moderate reduction in trait anxiety (Cohen’s d_rm = 0.39, 95% CI: 0.23–0.56; *p* < 0.001), indicating a statistically significant improvement in anxiety symptoms	Depression not assessed
Liu et al. (2024) [26]	PHQ-9; GAD-7; PSQI	Indirect improvement via sleep (*p* < 0.001), indicating reduced stress levels	Significant reduction (*p* < 0.001), indicating great improvement in anxiety	Significant reduction (*p* < 0.001), indicating great improvement in depression
Zheng et al. (2024) [40]	DASS	Significant within-group reduction (*p* < 0.01), but no statistically significant between-group differences	Significant within-group reduction (*p* < 0.01), but no statistically significant between-group differences	Significant within-group reduction (*p* < 0.01), but no statistically significant between-group differences

Note. PHQ-9 = Patient Health Questionnaire-9; BAI = Beck Anxiety Inventory; PSS = Perceived Stress Scale; FFMQ-SF = Five Facet Mindfulness Questionnaire–Short Form; CORE = Clinical Outcomes in Routine Evaluation; WEMWBS = Warwick–Edinburgh Mental Well-being Scale; HADS = Hospital Anxiety and Depression Scale; DASS-21 = Depression Anxiety Stress Scales–21 items; QoL = Quality of Life; STAI = State–Trait Anxiety Inventory; PANAS = Positive and Negative Affect Schedule; FMI = Freiburg Mindfulness Inventory; GAD-7 = Generalized Anxiety Disorder-7; PSQ-20 = Perceived Stress Questionnaire–20 items; BDI = Beck Depression Inventory; RRS = Ruminative Responses Scale; PSQI = Pittsburgh Sleep Quality Index; EMA = Ecological Momentary Assessment; WHO-5 = World Health Organization–5 Well-Being Index. Effect size/statistical indicators: B = unstandardized regression coefficient; β = standardized regression coefficient; d = Cohen’s d (effect size); g = Hedges’ g (effect size); MD = mean difference; R^2^ = coefficient of determination; *p* = *p*-value.

**Table 6:** Theoretical Foundations and Intervention Models of Mindfulness-Based Interventions (*n* = 24).

Author (Year)	Intervention	Theory/Framework/Model	Description
Ahmad et al. (2020) [1]	Mindfulness Virtual Community	Mindfulness and cognitive behavioral therapy	Web-based modules integrating CBT psychoeducation with mindfulness practice
Bai et al. (2020) [27]	Just Breathe (L2B adaptation)	Mindfulness-based emotion regulation	Teaches awareness of body, thoughts, and emotions, and aims to reduce reactivity in daily life
El Morr et al. (2020) [28]	Mindfulness Virtual Community	Mindfulness-based cognitive behavioral therapy	Online intervention integrating mindfulness practices, cognitive behavioral therapy, and virtual community support
Dorais and Gutierrez (2021) [29]	Headspace meditation app (app-based mindfulness)	Mindfulness meditation framework	App-based guided meditations are used at students’ discretion. Uses guided meditation to reduce distress and improve adjustment
Flett et al. (2020) [30]	Mindfulness Skills for Students	Mindfulness is based on cognitive and behavioral principles	Eight-week group course teaching meditation, inquiry, and reflective practices. Focuses on resilience, attention training, and emotional regulation
Galante et al. (2020) [14]	Qigong mind–body exercise	Traditional Chinese medicine and mindfulness	Slow movement, breathing, and meditative focus compared with the CBT relapse prevention protocol
Lu et al. (2020) [31]	Feel Stress Free app	Cognitive behavioral therapy	Digital CBT tools, including relaxation, thought challenging, and mood tracking
McCloud et al. (2020) [32]	BioBase app and wearable	Cognitive behavioral therapy, mindfulness, job demands, resources model	App delivers psychoeducation, breathing exercises, mood tracking, and biofeedback from wearable sensors
Ponzo et al. (2020) [33]	Centering meditation	Contemplative Christian tradition and spiritual meditation	Uses a sacred word to cultivate inner stillness and reduce stress
de Sousa et al. (2021) [34]	Brief mindfulness-based training	Mindfulness meditation	Short meditation-based intervention focusing on breath and body awareness
Karing and Beelmann (2021) [18]	Low-dose mindfulness training	Mindfulness is based on stress reduction	Uses multiple mindfulness practices to improve well-being
Ritvo et al. (2021) [35]	Mindfulness Virtual Community	Mindfulness-based cognitive behavioral therapy	Web-based program combining mindfulness practices, cognitive behavioral therapy principles, peer support, and guided videoconferencing
Rodriguez et al. (2021) [36]	Internet-based mindfulness	Mindfulness-based cognitive therapy	Combines mindfulness with cognitive strategies
Küchler et al. (2022) [25]	StudiCare Mindfulness	Acceptance and Commitment Therapy and Mindfulness-Based Stress Reduction	Five-module guided online program emphasizing mindfulness, cognitive defusion, values, and committed action
Küchler et al. (2023) [16]	StudiCare Mindfulness	Acceptance and Commitment Therapy and Mindfulness-Based Stress Reduction	A seven-module online program teaching mindfulness, acceptance, values, and stress management
Balci et al. (2023) [4]	Online mindfulness program	Acceptance and Commitment Therapy	Emphasizes psychological flexibility and mindfulness
Bolognino et al. (2023) [37]	MBM and LKM	Mindfulness process model	Ten-minute guided meditation targeting breath awareness or compassion
Gallo et al. (2023) [19]	Mindfulness-Based Intervention	Mindfulness-Based Relapse Prevention	Eight-week group program teaching awareness, acceptance, reactivity management, and skillful action for stress and mood regulation
Komariah et al. (2023) [38]	Mindfulness breathing meditation	Mindfulness-based stress reduction principles	Breath-focused meditation to reduce stress, anxiety, and depression
Balci et al. (2024) [17]	Online mindfulness program	Acceptance and Commitment Therapy	Adapted culturally for Turkish students to reduce stress
Barcaccia et al. (2024) [39]	Mindfulness-based intervention	Mindfulness-based stress reduction	Weekly guided meditation and daily breathing practice to reduce depression, rumination, and anxiety. Uses attention to the present moment, non-judgment, and breathing awareness
Ellison et al. (2024) [15]	Mindfulness-based cognitive therapy	Cognitive theory of anxiety and stress	Targets negative automatic thoughts and cognitive restructuring
Liu et al. (2024) [26]	Mindfulness-based cognitive therapy	Cognitive behavioral therapy and mindfulness	An eight-week MBCT program combining meditation with cognitive restructuring. Reduces rumination, increases awareness, and improves emotional regulation
Zheng et al. (2024) [40]	Mindfulness VR	Mindfulness and Attention Restoration Theory	Immersive VR nature scenes paired with guided mindfulness instructions

Note. CBT = Cognitive Behavioral Therapy; MBCT = Mindfulness-Based Cognitive Therapy; MBM = Mindfulness-Based Meditation; LKM = Loving-Kindness Meditation; VR = Virtual Reality.

**Table 7:** Comparison of In-Person vs. Digital Mindfulness Intervention.

Comparison Domain	In-Person Mindfulness Interventions	Digital Mindfulness Interventions
Overall evidence-based	Smaller but more structured body of evidence from face-to-face mindfulness/MBCT studies (Galante et al., 2020 [14]; de Sousa et al., 2021 [34]; Karing & Beelmann, 2021 [18]; Liu et al., 2024 [26]).	Larger and more diverse evidence base, including app-based, web-based, online, and smartphone-supported interventions (El Morr et al., 2020 [28]; Ritvo et al., 2021 [35]; McCloud et al., 2020 [32]; Flett et al., 2020 [30]; Küchler et al., 2022 [25]; Küchler et al., 2023 [16]; Balci et al., 2023 [4]; Balci et al., 2024 [17]; Rodriguez et al., 2021 [36]; Ellison et al., 2024 [15]; Ponzo et al., 2020 [33]; Gallo et al., 2023 [19]; Zheng et al., 2024 [40]; Bolognino et al., 2023 [37]; Pirbaglou et al., 2025 [41]).
Delivery structure	Usually scheduled, instructor-led, and standardized in pacing and attendance (Galante et al., 2020 [14]; Karing & Beelmann, 2021 [18]; Liu et al., 2024 [26]).	Usually flexible, remote, app- or web-based, and often self-paced or minimally guided (El Morr et al., 2020 [15,28]; McCloud et al., 2020 [32]; Flett et al., 2020 [30]; Ponzo et al., 2020 [33]; Ellison et al., 2024 [15]).
Guidance/support	Guidance is typically built into the intervention through direct instruction and structured participation (Galante et al., 2020 [14]; de Sousa et al., 2021 [34]; Karing & Beelmann, 2021 [18]).	Guidance varies considerably, from fully self-guided apps to guided online programs and peer-supported formats; support appears to improve engagement in some digital trials (Küchler et al., 2023 [16]; Rodriguez et al., 2021 [36]; Balci et al., 2024 [17]).
Engagement/adherence	Generally, more stable because of fixed schedules, attendance expectations, and implementation quality (Karing & Beelmann, 2021 [18]; Liu et al., 2024 [26]).	A recurrent challenge. Several digital studies reported low uptake, dropout, or variable completion, although supported formats improved adherence in some cases (Flett et al., 2020 [30]; [Balci et al., 2023 [4]; Küchler et al., 2023 [16]; Rodriguez et al., 2021 [36]).
Accessibility/flexibility	Lower flexibility because participation often depends on place, time, and instructor availability (Galante et al., 2020 [14]; Karing & Beelmann, 2021 [18]).	Stronger accessibility and convenience because students can participate remotely and often at their own pace (El Morr et al., 2020 [28]; McCloud et al., 2020 [32]; Ponzo et al., 2020 [33]; Balci et al., 2023 [4]; Ellison et al., 2024 [15]).
Scalability	More resource-intensive and harder to scale widely across large student populations (Galante et al., 2020 [14]; Liu et al., 2024 [26]).	More scalable and easier to disseminate broadly across campuses and hard-to-reach student groups (El Morr et al., 2020 [28]; Rodriguez et al., 2021 [36]; Balci et al., 2023 [4]; Pirbaglou et al., 2025 [41]).
Stress outcomes	Generally favorable in structured face-to-face interventions, though supported by fewer studies (Galante et al., 2020 [14]; de Sousa et al., 2021 [34]; [Liu et al., 2024 [26]).	Mixed but often positive; some studies reported clear stress reduction, whereas others showed stronger effects for anxiety, depression, mindfulness, or well-being than for stress specifically (Ritvo et al., 2021 [35]; El Morr et al., 2020 [28]; Ponzo et al., 2020 [33]; Ellison et al., 2024 [15]; Zheng et al., 2024 [40]).
Anxiety outcomes	Favorable reductions reported, especially in structured MBCT-type programs (de Sousa et al., 2021 [34]; [Liu et al., 2024 [26]).	One of the most consistently improved outcomes across digital studies (El Morr et al., 2020 [28]; McCloud et al., 2020 [32]; Küchler et al., 2022 [25]; Balci et al., 2023 [4]; Ellison et al., 2024 [15]; Zheng et al., 2024 [40]).
Depression outcomes	Strong effects may be more visible in high-symptom or clinical in-person samples (Liu et al., 2024 [26]).	Generally positive across several digital trials, although effect magnitude varies by platform, support, and adherence (El Morr et al., 2020 [28]; McCloud et al., 2020 [32]; Küchler et al., 2022 [16,32]; Barcaccia et al., 2024 [39]; Ponzo et al., 2020 [33]).
Mindfulness/well-being outcomes	Improvements are commonly seen in mindfulness and related process measures such as self-efficacy and body awareness (Karing & Beelmann, 2021 [18]; Galante et al., 2020 [14]).	Digital interventions appear especially useful for improving mindfulness, well-being, resilience, and broader functioning, even when symptom change is mixed (Flett et al., 2020 [30]; Balci et al., 2023 [4]; Küchler et al., 2023 [16]; Ponzo et al., 2020 [33]; Bolognino et al., 2023 [37]).
Follow-up/durability of effects	Evidence suggests a stronger potential for sustained benefits in some in-person studies (Galante et al., 2020 [14]; Karing & Beelmann, 2021 [18]).	Follow-up effects are reported in some studies, but the evidence is less consistent and often depends on continued engagement and completion (Flett et al., 2020 [30]; Küchler et al., 2023 [25]; Balci et al., 2024 [17]).
Main strengths	Structure, accountability, implementation quality, and potentially stronger continuity of practice (Galante et al., 2020 [14]; Karing & Beelmann, 2021 [18]; Liu et al., 2024 [26]).	Accessibility, privacy, scalability, convenience, and lower barriers to help-seeking (El Morr et al., 2020 [28]; Ponzo et al., 2020 [33]; Balci et al., 2023 [4]; Rodriguez et al., 2021 [36]).
Main limitations	Smaller evidence base, lower scalability, and dependence on staff, time, and space (Galante et al., 2020 [14]; Liu et al., 2024 [26]).	Engagement, adherence, and dropout remain the clearest recurring weaknesses; some digital interventions are feasible and acceptable without broad improvement across all outcomes (Flett et al., 2020 [30]; Balci et al., 2023 [4]; Küchler et al., 2023 [16]; Rodriguez et al., 2021 [36]).

**Table 8:** Methodological Quality Assessment (JBI RCT Checklist) (*n* = 24).

Author, Year, Country	Randomization	Allocation Concealment	Baseline Similarity	Participant Blinding	Provider Blinding	Assessor Blinding	Identical Treatment	Complete Follow-Up	Analysis by Groups	Same Measurement	Reliable Measurement	Appropriate Statistics	Appropriate Design	Overall Risk
Ahmad et al., 2020, [1], Canada	Yes	Yes	Yes	No	Unclear	Unclear	Yes	Yes	Yes	Yes	Yes	Yes	Yes	Low
Bai et al., 2020 [27], United States	Yes	Unclear	Yes	No	Unclear	Unclear	Yes	Yes	Yes	Yes	Yes	Yes	Yes	Moderate
El Morr et al., 2020 [28], Canada	Yes	Yes	Yes	No	Unclear	Unclear	Yes	Yes	Yes	Yes	Yes	Yes	Yes	Low
Flett et al., 2020 [30], NewZealand	Yes	Unclear	Yes	No	Unclear	Unclear	Yes	Yes	Yes	Yes	Yes	Yes	Yes	Moderate
Galante et al., 2020 [14], United Kingdom	Yes	Yes	Yes	No	Unclear	Unclear	Yes	No	Yes	Yes	Yes	Yes	Yes	Moderate
Lu et al., 2020 [31], China	Yes	Unclear	Yes	No	Unclear	Unclear	Yes	Yes	Yes	Yes	Yes	Yes	Yes	Moderate
McCloud et al., 2020 [32], United Kingdom	Yes	No	Yes	No	Unclear	Unclear	Yes	Yes	Unclear	Yes	Yes	Yes	Yes	Moderate
Ponzo et al., 2020 [33], United Kingdom	Yes	Yes	Yes	No	Unclear	Yes	Yes	Yes	Yes	Yes	Yes	Yes	Yes	Low
de Sousa et al., 2021 [34], Brazil	Yes	No	Yes	No	Unclear	Unclear	Yes	Yes	Unclear	Yes	Yes	Yes	Yes	Moderate
Dorais & Gutierrez, 2021 [29], United States	Yes	Unclear	Yes	No	Unclear	Unclear	Yes	Yes	Yes	Yes	Yes	Yes	Yes	Moderate
Karing & Beelmann, 2021 [18], Germany	Yes	Yes	Yes	No	Unclear	Unclear	Yes	Yes	Yes	Yes	Yes	Yes	Yes	Low
Ritvo et al., 2021 [35], Canada	Yes	Unclear	Yes	No	Unclear	Unclear	Yes	Yes	Yes	Yes	Yes	Yes	Yes	Moderate
Rodriguez et al., 2021 [36], China	Yes	Yes	Yes	No	Unclear	Unclear	Yes	Yes	Yes	Yes	Yes	Yes	Yes	Low
Küchler et al., 2022 [25], Germany, Austria, Switzerland	Yes	Unclear	Yes	No	Unclear	Unclear	Yes	Yes	Yes	Yes	Yes	Yes	Yes	Moderate
Balci et al., 2023 [4], Germany	Yes	Unclear	Yes	No	Unclear	Unclear	Yes	No	Yes	Yes	Yes	Yes	Yes	Moderate
Bolognino et al., 2023 [37], United States	Yes	Unclear	Yes	No	Unclear	Unclear	Yes	Yes	Yes	Yes	Yes	Yes	Yes	Moderate
Gallo et al., 2023 [19], Brazil	Yes	Yes	Yes	No	Unclear	Unclear	Yes	No	Yes	Yes	Yes	Yes	Yes	Moderate
Komariah et al., 2023 [38], Indonesia	Yes	Unclear	Yes	No	Unclear	Unclear	Yes	Yes	Yes	Yes	Yes	Yes	Yes	Moderate
Küchler et al., 2023 [16], Germany, Austria, Switzerland	Yes	Unclear	Yes	No	Unclear	Unclear	Yes	Yes	Yes	Yes	Yes	Yes	Yes	Moderate
Balci et al., 2024 [17], Turkey	Yes	Unclear	Yes	No	Unclear	Unclear	Yes	No	Yes	Yes	Yes	Yes	Yes	Moderate
Barcaccia et al., 2024 [39], Italy and New Zealand	Yes	Yes	Yes	No	Unclear	Unclear	Yes	Yes	Yes	Yes	Yes	Yes	Yes	Low
Ellison et al., 2024 [15], United States	Yes	Yes	Yes	No	Unclear	Unclear	Yes	Yes	Yes	Yes	Yes	Yes	Yes	Moderate
Liu et al., 2024 [26], China	Yes	Yes	Yes	No	Unclear	Unclear	Yes	Yes	Yes	Yes	Yes	Yes	Yes	Low
Zheng et al., 2024 [40], China	Yes	Unclear	Yes	No	Unclear	Unclear	Yes	Yes	Yes	Yes	Yes	Yes	Yes	Moderate

## Data Availability

No new data were created or analyzed in this study. Data sharing is not applicable to this article.

## References

[1] Ahmad F., El Morr C., Ritvo P., Othman N., Moineddin R. (2020). An eight-week, web-based mindfulness virtual community intervention for students’ mental health: Randomized controlled trial. JMIR Ment. Health.

[2] Roy S., Biswas A.K., Sharma M. (2025). Stress, anxiety, and depression as psychological distress among college and undergraduate students: A scoping review of reviews guided by the socio-ecological model. Healthcare.

[3] Roy S., Biswas A.K., Sharma M. (2026). Multilevel mental health determinants among college students: A social ecological scoping review. Ment. Health Prev..

[4] Balci S., Küchler A.-M., Ebert D.D., Baumeister H. (2023). An online mindfulness intervention for international students: A randomized controlled feasibility trial. Clin. Psychol. Eur..

[5] Healthy Minds Network Healthy Minds Study 2014 Data Report. https://healthymindsnetwork.org/wp-content/uploads/2019/04/HMS_national_DataReport_2014.pdf.

[6] Healthy Minds Network Healthy Minds Study 2023–2024 Data Report. https://healthymindsnetwork.org/wp-content/uploads/2024/09/HMS_national_report_090924.pdf.

[7] American College Health Association American College Health Association-National College Health Assessment III: Reference Group Executive Summary Spring 2024. https://www.acha.org.

[8] Center for Collegiate Mental Health 2024 Annual Report. https://ccmh.psu.edu.

[9] American Council on Education Key Mental Health Statistics in Higher Education. https://www.acenet.edu.

[10] Center for Collegiate Mental Health 2024 Annual Report, Publication No. STA 25-489. https://ccmh.psu.edu/assets/docs/CCMH%202024%20Annual%20Report.pdf.

[11] American Council on Education Key Mental Health Statistics in Higher Education: 2023–2024. https://www.acenet.edu/Documents/Mental-Health-Higher-Ed-Stats.pdf.

[12] Oliveira Carvalho P., Hülsdünker T., Carson F. (2021). The impact of the COVID-19 lockdown on European students’ negative emotional symptoms: A systematic review and meta-analysis. Behav. Sci..

[13] World Health Organization (2022). World Mental Health Report: Transforming Mental Health for All.

[14] Galante J., Stochl J., Dufour G., Vainre M., Wagner A.P., Jones P.B. (2021). Effectiveness of providing university students with a mindfulness-based intervention to increase resilience to stress: 1-year follow-up of a pragmatic randomised controlled trial. J. Epidemiol. Community Health.

[15] Ellison O.K., Bullard L.E., Lee G.K., Vazou S., Pfeiffer K.A., Baez S.E., Pontifex M.B. (2024). Examining efficacy and potential mechanisms of mindfulness-based cognitive therapy for anxiety and stress reduction among college students in a cluster-randomized controlled trial. Int. J. Clin. Health Psychol..

[16] Küchler A.-M., Schultchen D., Dretzler T., Moshagen M., Ebert D.D., Baumeister H. (2023). A three-armed randomized controlled trial to evaluate the effectiveness, acceptance, and negative effects of StudiCare Mindfulness, an Internet- and Mobile-Based Intervention for college students with no and “on demand” guidance. Int. J. Environ. Res. Public Health.

[17] Balci S., Küchler A.-M., Ebert D.D., Baumeister H. (2024). Culturally adapted Turkish version of an internet-based mindfulness intervention for university students: A randomized controlled feasibility trial. BMC Digit. Health.

[18] Karing C., Beelmann A. (2021). Evaluating the Implementation and Effectiveness of a Low-Dose Mindfulness-Based Intervention in a Student Sample: A Randomized Controlled Trial. Mindfulness.

[19] Gallo G.G., Curado D.F., Machado M.P.A., Espíndola M.I., Scattone V.V., Noto A.R. (2023). A randomized controlled trial of mindfulness: Effects on university students’ mental health. Int. J. Ment. Health Syst..

[20] Gao Y., Shi L., Fu N., Yang N., Weeks-Gariepy T., Mao Y. (2024). Mobile-Delivered Mindfulness Intervention on Anxiety Level Among College Athletes: Randomized Controlled Trial. J. Med. Internet Res..

[21] Wong S.Y., Chan J.Y., Zhang D., Lee E.K., Tsoi K.K. (2018). The safety of mindfulness-based interventions: A systematic review of randomized controlled trials. Mindfulness.

[22] Dawson A.F., Brown W.W., Anderson J., Datta B., Donald J.N., Hong K., Allan S., Mole T.B., Jones P.B., Galante J. (2020). Mindfulness-based interventions for university students: A systematic review and meta-analysis of randomised controlled trials. Appl. Psychol. Health Well-Being.

[23] Chen B., Yang T., Xiao L., Xu C., Zhu C. (2023). Effects of mobile mindfulness meditation on the mental health of university students: Systematic review and meta-analysis. J. Med. Internet Res..

[24] Zuo X., Tang Y., Chen Y., Zhou Z. (2023). The efficacy of mindfulness-based interventions on mental health among university students: A systematic review and meta-analysis. Front. Public Health.

[25] Küchler A.-M., Kahlke F., Vollbrecht D., Peip K., Ebert D.D., Baumeister H. (2022). Effectiveness, Acceptability, and Mechanisms of Change of the Internet-Based Intervention StudiCare Mindfulness for College Students: A Randomized Controlled Trial. Mindfulness.

[26] Liu W., Yuan J., Wu Y., Xu L., Wang X., Meng J., Wei Y., Zhang Y., Kang C.-Y., Yang J.-Z. (2024). A randomized controlled trial of mindfulness-based cognitive therapy for major depressive disorder in undergraduate students: Dose- response effect, inflammatory markers and BDNF. Psychiatry Res..

[27] Bai S., Elavsky S., Kishida M., Dvořáková K., Greenberg M.T. (2020). Effects of mindfulness training on daily stress response in college students: Ecological momentary assessment of a randomized controlled trial. Mindfulness.

[28] El Morr C., Ritvo P., Ahmad F., Moineddin R. (2020). Effectiveness of an 8-Week Web-Based Mindfulness Virtual Community Intervention for University Students on Symptoms of Stress, Anxiety, and Depression: Randomized Controlled Trial. JMIR Ment. Health.

[29] Dorais S., Gutierrez D. (2021). The Effectiveness of a Centering Meditation Intervention on College Stress and Mindfulness: A Randomized Controlled Trial. Front. Psychol..

[30] Flett J.A., Conner T.S., Riordan B.C., Patterson T., Hayne H. (2020). App-based mindfulness meditation for psychological distress and adjustment to college in incoming university students: A pragmatic, randomised, waitlist-controlled trial. Psychol. Health.

[31] Lu C., Zou L., Becker B., Griffiths M.D., Yu Q., Chen S.-T., Demetrovics Z., Jiao C., Chi X., Chen A. (2020). Comparative Effectiveness of Mind-Body Exercise Versus Cognitive Behavioral Therapy for College Students with Problematic Smartphone Use: A Randomized Controlled Trial. Int. J. Ment. Health Promot..

[32] McCloud T., Jones R., Lewis G., Bell V., Tsakanikos E. (2020). Effectiveness of a Mobile App Intervention for Anxiety and Depression Symptoms in University Students: Randomized Controlled Trial. JMIR Mhealth Uhealth.

[33] Ponzo S., Morelli D., Kawadler J.M., Hemmings N.R., Bird G., Plans D. (2020). Efficacy of the Digital Therapeutic Mobile App BioBase to Reduce Stress and Improve Mental Well-Being Among University Students: Randomized Controlled Trial. JMIR Mhealth Uhealth.

[34] de Sousa G.M., de Lima-Araújo G.L., de Araújo D.B., de Sousa M.B.C. (2021). Brief mindfulness-based training and mindfulness trait attenuate psychological stress in university students: A randomized controlled trial. BMC Psychol..

[35] Ritvo P., Ahmad F., El Morr C., Pirbaglou M., Moineddin R. (2021). A Mindfulness-Based Intervention for Student Depression, Anxiety, and Stress: Randomized Controlled Trial. JMIR Ment. Health.

[36] Rodriguez M., Eisenlohr-Moul T.A., Weisman J., Rosenthal M.Z. (2021). The Use of Task Shifting to Improve Treatment Engagement in an Internet-Based Mindfulness Intervention Among Chinese University Students: Randomized Controlled Trial. JMIR Form. Res..

[37] Bolognino S.J., Renshaw T.L., Phan M.L. (2023). Differential effects of mindful breathing and loving kindness meditations: A component analysis study. Adv. Ment. Health.

[38] Komariah M., Ibrahim K., Pahria T., Rahayuwati L., Somantri I. (2023). Effect of Mindfulness Breathing Meditation on Depression, Anxiety, and Stress: A Randomized Controlled Trial among University Students. Healthcare.

[39] Barcaccia B., Medvedev O.N., Pallini S., Mastandrea S., Fagioli S. (2024). Examining mental health benefits of a brief online mindfulness intervention: A randomised controlled trial. Mindfulness.

[40] Zheng L., Li W., Song S., Xiao X., Low S.R., Zhang Y., Yu X., Peng Y. (2024). Effectiveness of Mindfulness-Based Virtual Reality Training on Stress, Anxiety, and Depression Among Chinese University Students. Mindfulness.

[41] Pirbaglou M., El Morr C., Ahmad F., Ritvo P. (2025). Mindful Nonreactivity, Anxiety, Depression, and Perceived Stress as Mediators of the Mindfulness Virtual Community Intervention—Pathways to Enhance Mental Health in University Students: Secondary Evaluation of Two Randomized Controlled Trials with Student Participants. JMIR Ment. Health.

[42] Joanna Briggs Institute JBI Manual for Evidence Synthesis. https://synthesismanual.jbi.global.

[43] Page M.J., McKenzie J.E., Bossuyt P.M., Boutron I., Hoffmann T.C., Mulrow C.D., Shamseer L., Tetzlaff J.M., Akl E.A., Brennan S.E. (2021). The PRISMA 2020 statement: An updated guideline for reporting systematic reviews. BMJ.

[44] Roy S., Biswas A.K., Sharma M. (2025). Evidence-Based Clinical Effectiveness of Kundalini Yoga: Systematic Review of RCTs Across Multiple Health Conditions. Altern. Ther. Health Med..

